# An integrative redescription of the nominal taxon for the *Mesobiotus harmsworthi* group (Tardigrada: Macrobiotidae) leads to descriptions of two new *Mesobiotus* species from Arctic

**DOI:** 10.1371/journal.pone.0204756

**Published:** 2018-10-17

**Authors:** Łukasz Kaczmarek, Krzysztof Zawierucha, Jakub Buda, Daniel Stec, Magdalena Gawlak, Łukasz Michalczyk, Milena Roszkowska

**Affiliations:** 1 Department of Animal Taxonomy and Ecology, Faculty of Biology, Adam Mickiewicz University, Poznań, Poznań, Poland; 2 Department of Entomology, Institute of Zoology and Biomedical Research, Jagiellonian University, Kraków, Poland; 3 The Institute of Plant Protection-National Research Institute, Poznań, Poland; 4 Department of Bioenergetics, Faculty of Biology, Adam Mickiewicz University, Poznań, Poznań, Poland; Ciimar, PORTUGAL

## Abstract

The *Mesobiotus harmsworthi* group has a global distribution, with localities in polar, temperate and tropical zones. Since the first species of the *harmsworthi* group was described in the beginning of the 20^th^ century, tens of new species within the group were found and named. However, the diagnosis of the nominal *Mesobiotus harmsworthi* is insufficient and enigmatic, thus it can be is a serious obstacle in solving the taxonomy of this group. Here, we integratively redescribe the nominal species for the genus *Mesobiotus*, *i*.*e*., *Mesobiotus harmsworthi* and clarify taxonomic statuses of the two subspecies: *M*. *harmsworthi harmsworthi* and *M*. *harmsworthi obscurus* that have been recognised as distinct taxa for more than three decades. Traditionally, egg chorion in *M*. *harmsworthi* was considered almost smooth and without any traces of areolation, however here we report many misunderstandings that accumulated across decades and we show that, in fact, the chorion in this species exhibits a partially developed areolation. We present an integrative (morphological, morphometric and molecular) diagnosis of the nominal taxon and we confirm that it differs from other species of the *harmsworthi* group by morphological characters of both animals and eggs. Additionally, we describe two new species of the genus *Mesobiotus*: *M*. *skorackii* **sp. nov.** from the Kyrgyz Republic (using classical morphological description) and *M*. *occultatus* **sp. nov.** from Svalbard Archipelago (by means of integrative taxonomy). Finally, we also provide the first genetic phylogeny of the genus *Mesobiotus* based on COI sequences which, together with molecular species delimitation, independently confirms the validity of the analysed taxa.

## Introduction

The phylum Tardigrada comprises over 1,200 species [1, 2, 3] that inhabit both aquatic (freshwater, brackish, marine) and terrestrial environments throughout the world, from the deepest seas to the highest mountain peaks [4, 5, 6, 7]. Tardigrades have been investigated for over two hundred years, but because of old descriptions and insufficient morphometric data, many species currently need revision and redescription, especially those representing nominal taxa for cosmopolitan groups or genera. One of such taxa is *Mesobiotus* Vecchi, Cesari, Bertolani, Jönsson, Rebecchi & Guidetti, 2016 [8], a cosmopolitan genus comprising *ca*. 40 known species that inhabit plants, lichens and soil and are considered carnivorous and/or omnivorous [8].

The genus *Mesobiotus* is divided into two species groups: the *harmsworthi* and the *furciger* group, classified within the genus *Macrobiotus* C.A.S. Schultze, 1834 [9] prior to the erection of *Mesobiotus*. Species of the *harmsworthi* group are characterised by three clearly separated macroplacoids in the shape of short, rounded rods and a distinct microplacoid situated very close to them, as well as by conical or hemispherical egg processes [10]. *Mesobiotus harmsworthi* (Murray, 1907) [11], the nominal species for the *harmsworthi* group, was described by Murray [11] as *Macrobiotus harmsworthi*. Since the original description, the problem with the exact characteristics of the species was addressed several times by different authors (e.g. [4, 12, 13]). Moreover, descriptions of two *M*. *harmsworthi* subspecies, *M*. *harmsworthi coronatus* (de Barros, 1942) [14] and *M*. *harmsworthi obscurus* (Dastych, 1985) [15], did complicate the taxonomy of this group even further. The first subspecies was later elevated to the species level by Pilato et al. [13], whereas the second is considered valid as a subspecies. Although problems with the taxonomy of the nominal species were broadly discussed in literature, a formal redescription of *M*. *h*. *harmsworthi*, based on type material or material from the type locality, has not been published. The only attempt to characterise *M*. *h*. *harmsworthi* in more detail was made by Pilato et al. [13]. However, the work was based solely on light microscope observations, thus some fine morphological details, that are below light microscope resolution, could remain unknown. Also, molecular markers, that are necessary for the accurate characterisation of the species and for the detection of potential cryptic species, are not available. Furthermore, in addition to specimens from *loci typici* (Spitsbergen and Shetlands), Pilato et al. [13] pooled and analysed together individuals and eggs from Italy and France. Thus, without DNA sequences, it is not possible to verify whether all specimens constituted a single or multiple cryptic or pseudocryptic species.

Therefore, in order to clarify the taxonomy of the *harmsworthi* group, we integratively redescribe *M*. *h*. *harmsworthi* based on typical material of *M*. *h*. *obscurus* (which is now, in fact, a synonym of *M*. *h*. *harmsworthi s*.*s*.; for more details see Results and Discussion sections below) and we additionally describe two new species of the *harmsworthi* group: *M*. *occultatus* **sp. nov.** from Spitsbergen and *M*. *skorackii* **sp. nov.** from the Kyrgyz Republic. Our study involved an integrative taxonomy approach by combing morphological and morphometric data from phase contrast light microscopy (PCM) and molecular data in form of DNA sequences of four molecular markers (nuclear: 18S rRNA, 28S rRNA, ITS-2 and mitochondrial: COI). Finally, we provide the first COI phylogeny of the genus *Mesobiotus*.

## Material and methods

### Sample processing

Moss and lichen samples with *M*. *h*. *harmsworthi*, *M*. *occultatus* **sp. nov.** and *M*. *skorackii* **sp. nov.** were collected by ŁK and KZ during scientific expeditions to the Kyrgyz Republic in July 2002, to Spitsbergen in June–July 2010, August 2011 and to Spitsbergen and Sjuøyane (Phippsøya) in 2013 (Table 1). All samples were air-dried in paper envelopes and delivered to the laboratory at the Faculty of Biology, Adam Mickiewicz University in Poznań. Tardigrades were extracted, mounted on microscope slides in Hoyer’s medium and studied following the protocol by Dastych [16] with modifications by Stec et al. [17]. Additionally, two unidentified *Mesobiotus* species, one of the *furciger* group collected from continental Norway (Lyngen Alps, moraine of Steindalen glacier; 69°23'44.04"N, 19°55'0.84"E) and the other of the *harmsworthi* group collected from Russia (Irkuck; 52°16'42.3''N, 104°17'22.1''E), were extracted from moss samples and then used in phylogenetic analysis.

**Table 1:** The list of *Mesobiotus* specimens from Svalbard archipelago genotyped for the phylogenetic analysis with their collection details (numbers in brackets indicate number of sequences and number of studied specimens).

Species (No of specimens)	Coordinates	Locality	Sample details	DNA marker (No of sequences)
*M*. *harmsworthi s*.*s*.(4)	77°00'47''N; 15°31'12''E	Spitsbergen	moss on rock, 300 m asl	ITS-2 (2), COI (3), 28S rRNA (1), 18S rRNA (2)
*M*. *harmsworthi s*.*s*.(2)	80°41'13''N; 20°50'40''E	Phippsøya	moss on rock, 47 m asl	COI (2)
*M*. *occultatus* **sp. nov.** (4)	77°00'48''N; 15°33' 05''E	Spitsbergen	moss on rock, 11 m asl	ITS-2 (3), COI (2), 18S rRNA (2)

### Microscopy and imaging

All measurements and photomicrographs were taken using an Olympus BX41 phase contrast microscope (PCM) associated with an ARTCAM-300Mi digital camera (Olympus Corporation, Shinjuku-ku, Japan). In order to obtain clean and fully extended material for SEM, animals and eggs were processed according to Roszkowska et al. [18]. In brief, specimens were first heated to *ca*. 70°C in distilled water in order to obtain stretched animals, then rinsed several times with ddH_2_O, then subjected to a water/ethanol series (from 0% to 100% ethanol, with 10% increments), then to one ethanol/acetone series (100% ethanol and 100% acetone in 1:1 proportion), and at the end three times rinsed with 100% acetone. The material was transferred between solutions in small cages made of a short plastic tube closed at the ends with fine plastic mesh (Ø 40 μm). The dehydrated specimens and eggs were then dried at the CO_2_ critical point, transferred with an eyebrow hair mounted on a wooden stick onto a SEM stub covered with double-sided conductive tape, and sputter coated with a thin layer of gold. Specimens were examined under high vacuum in a Hitachi S3000N Scanning Electron Microscope at the Institute of Plant Protection in Poznań.

All figures were assembled in Corel Photo-Paint 9. For deep structures that could not be fully focused in a single photograph, a series of 2–10 images were taken and then assembled into a single deep-focus image manually in Corel Photo-Paint 9.

### Morphometrics and morphological nomenclature

All measurements, made with the QuickPhoto Camera 2.3 software, are given in micrometres [μm]. Structures were measured only if their orientation was suitable. Body length was measured from the anterior extremity to the end of the body, excluding the hind legs. The types of bucco-pharyngeal apparatuses and claws were classified according to Pilato & Binda [19] and Vecchi et al. [8]. The terminology used to describe oral cavity armature, and used in differential diagnoses, follows Michalczyk & Kaczmarek [20]. Buccal tube length and the level of the stylet support insertion point were measured according to Pilato [21]. Other buccal apparatus traits and claws were measured according to Kaczmarek & Michalczyk [22]. Macroplacoid length sequence is given according to Kaczmarek *et al*. [23]. The *pt* ratio is the ratio of the length of a given structure to the length of the buccal tube expressed as a percentage [21]. Distance between egg processes was measured as the shortest line connecting base edges of the two closest processes [22]. Morphometric data were handled using the “Parachela” ver. 1.3 template available from the Tardigrada Register [24]. Tardigrade taxonomy follows Bertolani *et al*. [25] and Vecchi et al. [8].

### Comparative material

**Fig 1:**
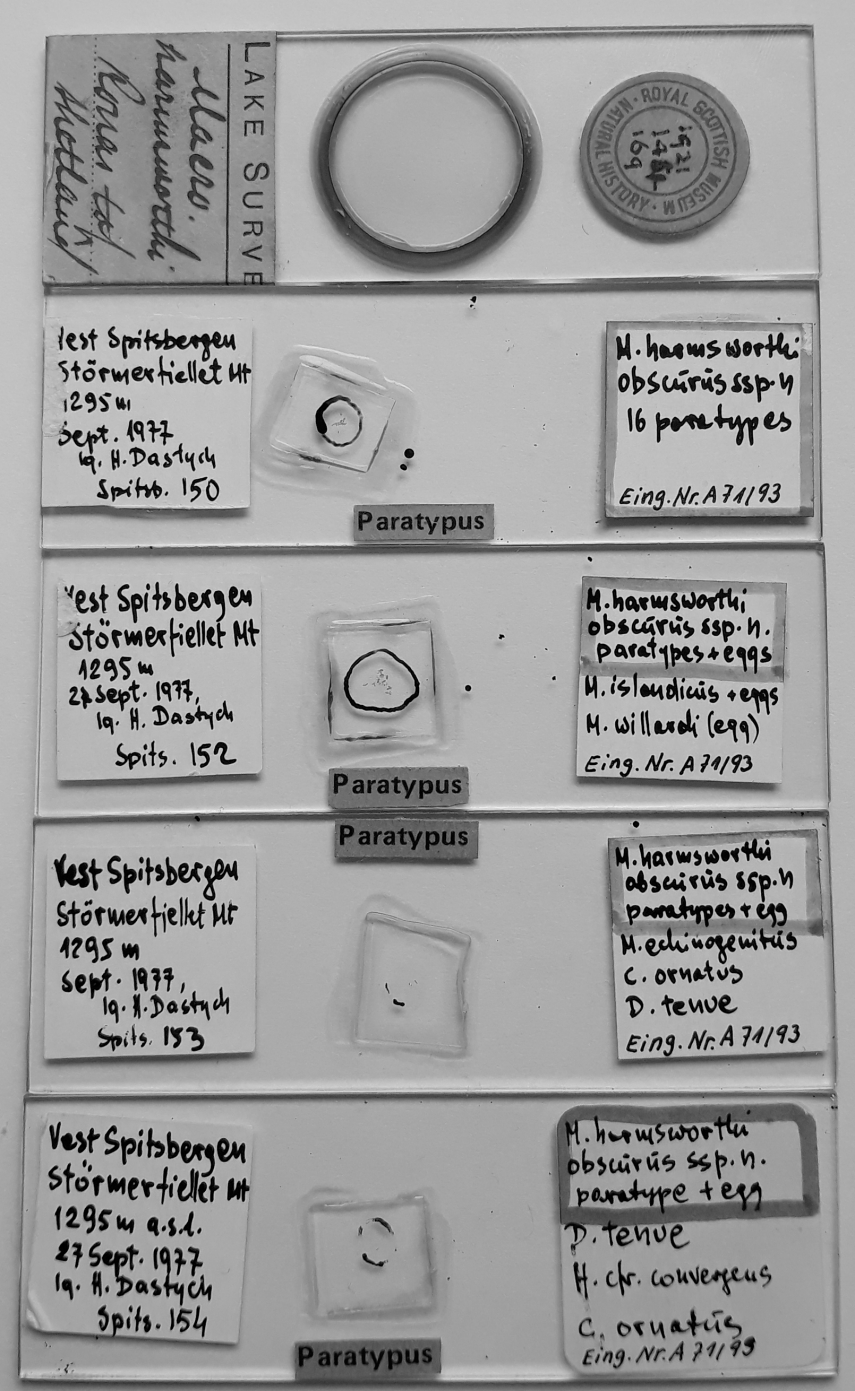
Examined microscope slides: With “*M*. *harmsworthi*” from the Murray collection deposited at the National Museum of Scotland in Edinburgh, and with paratypes of *M*. *h*. *obscurus* deposited at the Zoological Museum of the Hamburg University (ZMHU).

Paratypes of *M*. *h*. *obscurus* (for details see redescription of *M*. *harmsworthi*, below) were borrowed from Zoological Museum of Hamburg University (ZMHU). A single specimen of *M*. *harmsworthi* (labelled as: “Z.1921.144.169, *Macro*. *harmsworthi*, Ronas Top, Shetland”) from the Murray collection was borrowed from the National Museum of Scotland in Edinburgh (Fig 1). The species were identified based on the key in Kaczmarek et al. [10] and original descriptions [13, 14, 15, 26, 27, 28, 29, 30, 31, 32, 33]. Additionally, holotypes and paratypes of *M*. *barbarae* (Kaczmarek, Michalczyk & Degma, 2007 [32]), *M*. *binieki* (Kaczmarek, Gołdyn, Prokop & Michalczyk, 2011 [10]), *M*. *ethiopicus* Stec & Kristensen, 2017 [34], *M*. *insanis* Mapalo, Stec, Mirano-Bascos & Michalczyk, 2017 [35], *M*. *philippinicus* Mapalo, Stec, Mirano-Bascos & Michalczyk, 2016 [36], *M*. *pseudoblocki* Roszkowska, Stec, Ciobanu & Kaczmarek, 2016 [37], *M*. *pseudopatiens* Kaczmarek & Roszkowska, 2016 [38], *M*. *reinhardti* (Michalczyk & Kaczmarek, [20]), and *M*. *szeptyckii* (Kaczmarek & Michalczyk, [39]) were examined under PCM.

### Genotyping

For DNA isolation and sequencing, three individuals of *M*. *harmsworthi* and five individuals of *M*. *occultatus* **sp. nov.** were used (Table 1). The list of individuals with their collection details are provided in Table 2. The genomic DNA was extracted from single individuals according to the protocol described in Dabert et al. [40], using Tissue Kit (Qiagen GmbH, Hilden, Germany). In order to obtain tardigrade exoskeletons, a technique adopted by Zawierucha *et al*. [41] was used. Specifically, individuals were digested for 48 hours (56°C) in mixture of ATL buffer, proteinase K in Eppendorf vials and then centrifuged at 6000 rpm. From each vial 90 μm of DNA extract was taken for further analyses by carefully removing this volume using a half-automatic pipette. The remaining 10 μm of the DNA extract with the tardigrade exoskeleton was preserved in 96% ethanol. Then, exoskeletons were mounted in Hoyer’s medium for PCM analyses.

**Table 2:** Primers and references for PCR programmes used for sequencing of DNA fragments.

DNA fragment	Primer name	Primer direction	Primer sequence (5’-3’)	Source	Program
18S rRNA	18sFw	Forward	CTTGTCTCAAAGATTAAGCCATGCA	[87]	[88]
	18srev930	Reverse	GACGGTCCAAGAATTTCAC		
	18S_Tar_Ff1	Forward	AGGCGAAACCGCGAATGGCTC	[34]	
	18S_Tar_Rr1	Reverse	GCCGCAGGCTCCACTCCTGG		
28S rRNA	28sF0001	Forward	ACCCVCYNAATTTAAGCATAT	[89]	[89]
	28sR0990	Reverse	CCTTGGTCCGTGTTTCAAGAC		
ITS-2	ITS2_Eutar_Ff	Forward	CGTAACGTGAATTGCAGGAC	[90]	[90]
	ITS2_Eutar_Rr	Reverse	TGATATGCTTAAGTTCAGCGG		
COI	bcdF01	Forward	CATTTTCHACTAAYCATAARGATATTGG	[87]	[87]
	bcdR04	Reverse	TATAAACYTCDGGATGNCCAAAAAA		
	LCO1490	Forward	GGTCAACAAATCATAAAGATATTGG	[91]	[85]
	HCO2198	Reverse	TAAACTTCAGGGTGACCAAAAAATCA		

Further molecular analyses involved the amplification and sequencing of four DNA markers, three nuclear (18S rRNA, 28S rRNA, ITS-2) and one mitochondrial (COI). The markers differ in effective mutation rates: the first two are considered conservative whereas the last two are more variable. The 18S rRNA together with 28S rRNA are often used for resolving relationships at higher taxonomic levels such as families and genera (e.g. [25]) whereas COI and ITS-2 are appropriate for examining intraspecific and intrageneric genetic variation (e.g. [42, 43, 44, 45, 46, 47]).

Amplification of DNA fragments (PCR) for 18S rRNA and 28S rRNA was conducted in the total volume of 5.5 μl including: 3 μl Type-it Microsatellite PCR Kit (Qiagen), 0.5 μl of each primer, 0.5 μl Q-Solution (Qiagen) and 1 μl of the DNA template. For ITS-2 and COI a total volume of PCR mix was 5 μl including: 3 μl Type-it Microsatellite PCR Kit (Qiagen), 0.5 μl of each primer and 1 μl of the DNA template. Each PCR reactions proceeded in the following steps: One cycle of 5 min. at 95°C, followed by 40 steps of 30 s at 95°C, 90 s at 50°C, 1 min at 72°C, and with a final elongation step of 5 min at 72°C. After PCRs each products were diluted by 5 μl MQ water. Separation of PCR products were carried out by 1% agarose gel electrophoresis. Samples containing visible bands were purified with exonuclease I and Fast alkaline phosphatase (Fermentas). The fragments were sequenced using the BigDye Terminator v3.1 kit and the ABI Prism 3130xl Genetic Analyzer (Applied Biosystems), following manufacturer instructions.

All four mentioned above molecular markers were sequenced for two additional *Mesobiotus* species from Norway and Russia following the protocol by Stec et al. [17]. Only for 18S rRNA and COI other primers were used: 18S_Tar_Ff1 with 18S_Tar_Rr1 and LCO1490 with HCO2189, respectively (see Table 2 for details).

### Comparative molecular analysis

For molecular comparisons, all published sequences of the four abovementioned markers for the genus *Mesobiotus* were downloaded from GenBank (listed in Table 3). The sequences were aligned using the default settings (for COI) and the Q-INS-I method (for ribosomal markers: 18S rRNA, 28S rRNA and ITS-2) of MAFFT version 7 [48, 49] and manually checked against non-conservative alignments in BioEdit. Then, the aligned sequences were trimmed to: 721 (18S rRNA), 751 (28S rRNA), 497 (ITS-2), 565 (COI) bp. All COI sequences were translated into protein sequences in MEGA7 version 7.0 [50] to check against pseudogenes. Genetic distances were calculated using MEGA7 as suggested by Srivathsan & Meier [51], *i*.*e*., as uncorrected pairwise distances instead of K2P distances. The matrices with calculated uncorrected p-genetic distances for each of the analysed DNA fragment are provided as supplementary materials (S1 Table).

**Table 3:** Sequences used for molecular comparisons between the *Mesobiotus* species redescribed/described in this study and all other species of the genus *Mesobiotus*, for which homologous DNA sequences are currently available. The 18S rRNA sequence of *M*. *insanis* has not been used because of its shortness. The sequences with underline GenBank accession numbers were included in the concatenated data matrix to construct the phylogenetic tree (Fig 18) whereas bolded numbers indicate new sequences obtained in this study.

DNA marker	Species	Accession number	Source
18S rRNA	*M*. *ethiopicus*	MF678793	[34]
	*M*. *philippinicus*	KX129793	[35]
	*M*. *hilariae* Vecchi et al., 2016 [8]	KT226068-71	[8]
	*M*. *polaris* (Murray, 1910)	KT226075-78	[8]
	*M*. *cf*. *mottai*	KT226072	[8]
	*M*. *harmsworthi* group species	KT226073-74	[8]
	*M*. *radiatus*	MH197153	[92]
	*M*. *romani* Roszkowska et al., 2018	MH197158	[18]
	*M*. *furciger* group species	**MH197148**	this study
	*M*. *harmsworthi* group species	**MH197149**	this study
	*M*. *harmsworthi* group species	HQ604967-70	[25]
	*M*. *furciger* (Murray, 1907)	EU266927-28	[93]
28S rRNA	*M*. *ethiopicus* Stec and Kristensen, 2017	MF678792	[34]
	*M*. *philippinicus* Mapalo et al., 2016	KX129794	[36]
	*M*. *insanis* Mapalo et al., 2017	MF441489	[35]
	*M*. *radiatus* (Pilato et al., 1991)	MH197152	[92]
	*M*. *romani* Roszkowska et al., 2018	MH197151	[18]
	*M*. *furciger* group species	**MH197265**	this study
	*M*. *harmsworthi* group species	**MH197266**	this study
ITS-2	*M*. *philippinicus* Mapalo et al., 2016	KX129795	[36]
	*M*. *insanis* Mapalo et al., 2017	MF441490	[35]
	*M*. *radiatus* (Pilato et al., 1991)	MH197267	[92]
	*M*. *romani* Roszkowska et al., 2018	MH197150	[18]
	*M*. *furciger* group species	**MH197156**	this study
	*M*. *harmsworthi* group species	**MH197157**	this study
COI	*M*. *ethiopicus* Stec and Kristensen, 2017	MF678794	[34]
	*M*. *philippinicus* Mapalo et al., 2016	KX129796	[36]
	*M*. *insanis* Mapalo et al., 2017	MF441491	[35]
	*M*. *hilariae* Vecchi et al., 2016	KT226108	[8]
	*M*. *radiatus* (Pilato et al., 1991)	MH195147	[92]
	*M*. *romani* Roszkowska et al., 2018	MH195149	[18]
	*M*. *furciger* group species	**MH195153**	this study
	*“M*. *harmsworthi”*	GU113140	unpublished
	*M*. *harmsworthi* group species	**MH195154**	this study
	*M*. *furciger* (Murray, 1907)	JX865306, JX865308, JX865314	[94]

### Phylogenetic and species delimitation analysis

To establish phyletic relationships of *M*. *harmsworthi* and *M*. *occultatus* **sp. nov.** and to molecularly delineate the species, we constructed a phylogenetic tree based on all COI sequences for the genus *Mesobiotus* available from GenBank (Table 3) together with sequences obtained in the present study. The COI sequences were aligned using the default settings of MAFFT version 7 [48, 49]. The obtained alignment was edited and checked manually in BioEdit and then trimmed to 565 bp. The COI sequence of *Macrobiotus scoticus* Stec, Morek, Gąsiorek, Blagden & Michalczyk, 2017 [52] (GenBank accession number KY797267) and *Macrobiotus shonaicus* Stec, Arakawa & Michalczyk, 2018 [53] (MG757136–7) were used as outgroups. Given that COI is a protein coding gene, before partitioning, we divided our alignment into three data blocks constituting separated three codon positions. Using PartitionFinder version 2.1.1 [54] under the Bayesian Information Criterion (BIC), the best scheme of partitioning and substitution models were chosen for posterior phylogenetic analysis. We ran the analysis to test all possible models implemented in the program. As best-fit partitioning scheme, PartitionFinder suggested to retain three predefined partitions separately. The best fit-models for these partitions were: SYM+I+G for the first codon position, GTR+I for the second codon position and TVM+I+G for the third codon position. Bayesian phylogenetic tree obtained from this data set was highly polytomous, thus we used only for molecular species delimitation analysis with the PTP method, which uses a non-ultrametric phylogenetic tree as the input data, based on which, the switch from speciation to coalescent processes is modelled and then used to delineate species [55]. For the purpose of the PTP analysis, we discarded the outgroup to protect against eventual biases caused by the distant relationship between the outgroup and the ingroup taxa. The calculations were conducted on the bPTP webserver (http://species.h-its.org/ptp), with 100,000 MCMC generations, thinning the set to 100, burning at 10% and performing search for Maximum Likelihood and Bayesian solutions. Then, in order to reduce polytomy and search for more resolved relationships, we concatenated the COI data set with three nuclear markers, 18S rRNA, 28S rRNA and ITS-2, using SequenceMatrix [56]. Since the molecular data on the genus *Mesobiotus* are limited not all taxa have been represented in all added data sets (see Table 3 for details). Before concatenation, we aligned sequences of each nuclear marker using the Q-INS-I method of MAFFT version 7, which considers the secondary structure of ribosomal genes [48, 49]. Next, we manually checked against non-conservative alignments in BioEdit. The aligned sequences were trimmed to: 721 (18S rRNA), 751 (28S rRNA) and 497 (ITS-2). Before partitioning, we divided our alignment into 6 data blocks constituting three separate blocks of ribosomal markers and three separate blocks of three codon positions in COI data set. Using PartitionFinder under the Bayesian Information Criterion (BIC), the best scheme of partitioning and substitution models were chosen for posterior phylogenetic analysis. We ran the analysis to test all possible models implemented in the program. As best-fit partitioning scheme, PartitionFinder suggested to retain six predefined partitions separately. The best fit-models for these partitions were: K80+G for 18S rRNA, SYM+G for 28S rRNA, K80+G for ITS-2, GTR+I+G for the first and the third codon position, and HKY+G for the second codon position.

Bayesian inference (BI) marginal posterior probabilities were calculated for both the COI and the concatenated (COI+18S rRNA+28S rRNA+ITS-2) data set using MrBayes v3.2 [57]. Random starting trees were used and the analysis was run for ten million generations, sampling the Markov chain every 1000 generations. An average standard deviation of split frequencies of <0.01 was used as a guide to ensure the two independent analyses had converged. The program Tracer v1.6 [58] was then used to ensure Markov chains had reached stationarity, and to determine the correct ‘burn-in’ for the analysis which was the first 10% of generations. The ESS values were greater than 200 and the consensus tree was obtained after summarising the resulting topologies and discarding the ‘burn-in’. The consensus tree was viewed and visualised by FigTree v.1.4.3 available from http://tree.bio.ed.ac.uk/software/figtree.

### Nomenclatural acts

The electronic edition of this article conforms to the requirements of the amended International Code of Zoological Nomenclature, and hence the new names contained herein are available under that Code from the electronic edition of this article. This published work and the nomenclatural acts it contains have been registered in ZooBank, the online registration system for the ICZN. The ZooBank LSIDs (Life Science Identifiers) can be resolved and the associated information viewed through any standard web browser by appending the LSID to the prefix "http://zoobank.org/". The LSID for this publication is: urn:lsid:zoobank.org:pub:86D87D80-5398-4D89-9767-C8E2A160ADFE. The electronic edition of this work was published in a journal with an ISSN, and has been archived and is available from the following digital repositories: PubMed Central, LOCKSS.

## Results

### Taxonomic account

**Phylum:** Tardigrada Doyère, 1840 [59]

**Class:** Eutardigrada Richters, 1926 [60]

**Order:** Parachela Schuster, Nelson, Grigarick & Christenberry, 1980 [61]

**Superfamily:** Macrobiotoidea Thulin, 1928 [62] (in Marley et al. 2011) [63]

**Family:** Macrobiotidae Thulin, 1928 [62]

**Genus:** *Mesobiotus* Vecchi, Cesari, Bertolani, Jönsson, Rebecchi & Guidetti, 2016 [8]

### *Mesobiotus harmsworthi* (Murray, 1907) [11]

*M*. *harmsworthi*, sp. n. [11]

*Macrobiotus* harmsworthi obscurus ssp. nov. [15]

**Fig 2:**
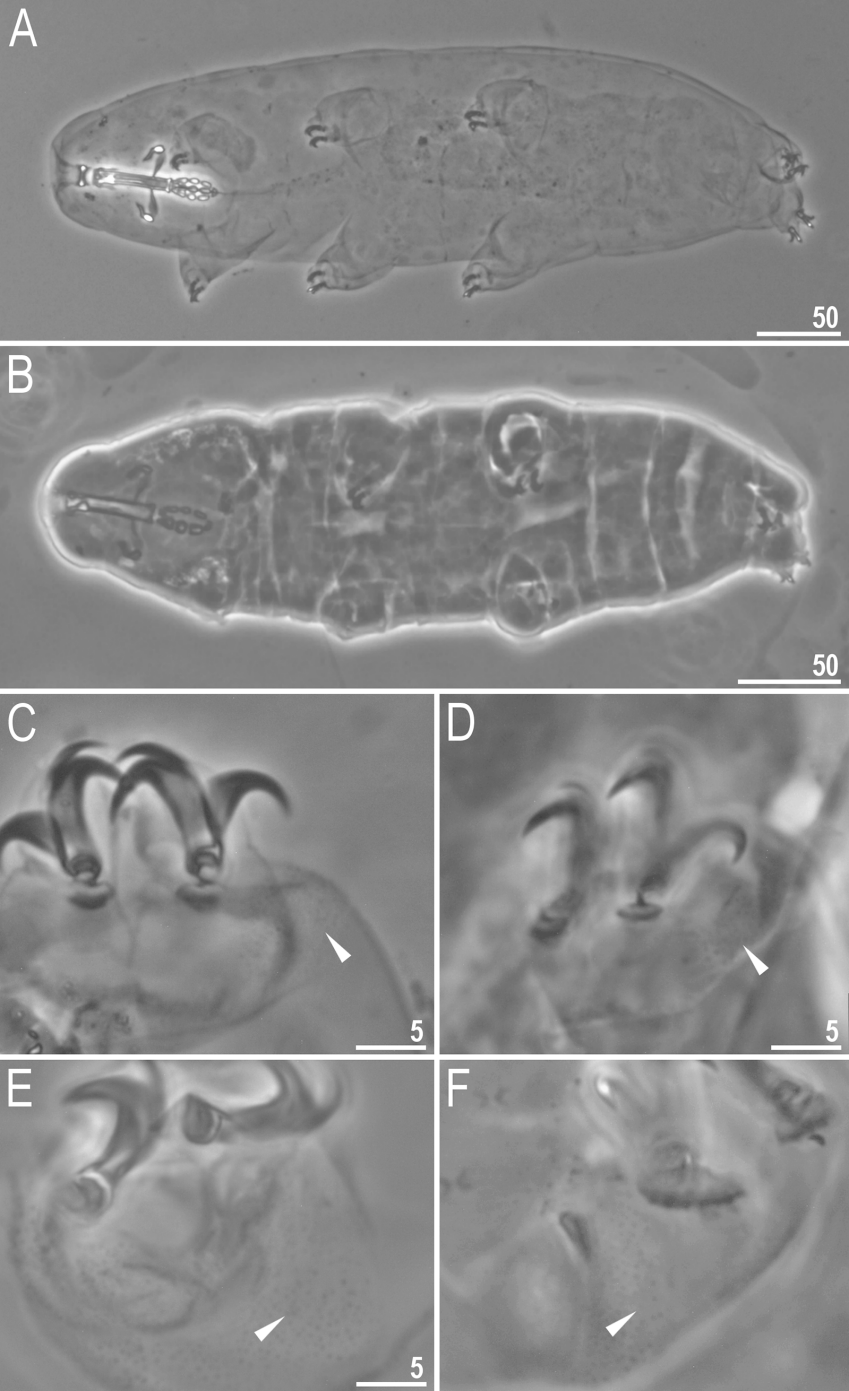
*Mesobiotus harmsworthi*–habitus and granulation on legs. A–B–dorso-ventral projection of the entire animal; C–D–granulation on leg III and II, respectively, arrowheads; E–F–granulation on leg IV, arrowheads. Scale bars in micrometres [μm]. All PCM. B, D, F–*Macrobiotus harmsworthi obscurus* ssp. nov. (Dastych 1985) (= *Mesobiotus harmsworthi* s.s. (Murray, 1907)–photos of paratypes from ZMHU.

**Fig 3:**
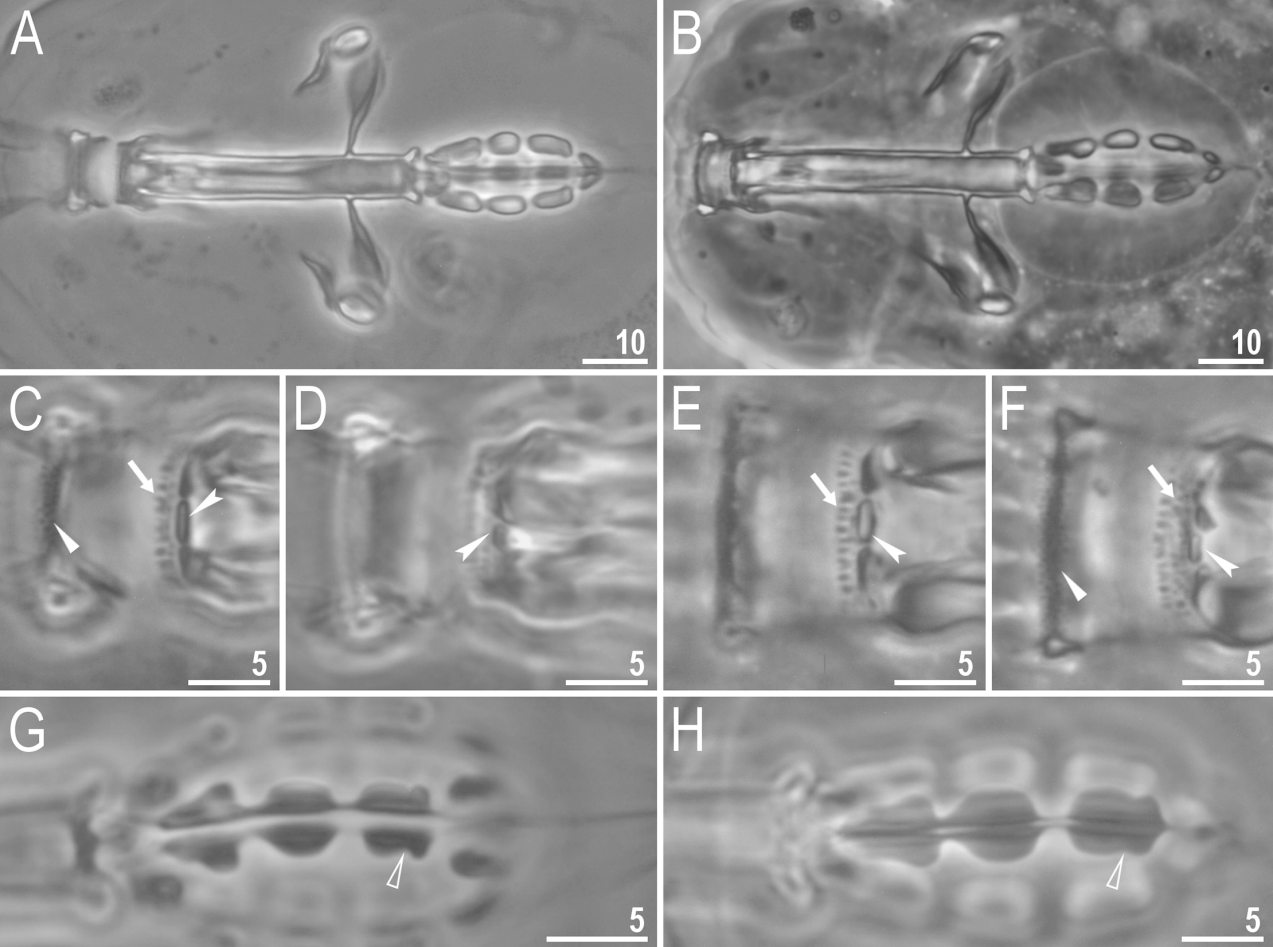
*Mesobiotus harmsworthi*—buccal apparatus and the oral cavity armature. A–B–general view; C–F–oral cavity armature; flat arrowheads indicate teeth of the first band, arrows indicate teeth of the second band, indented arrowheads indicate teeth of the third band; G–H–ventral placoids; empty arrowheads indicate a subterminal constriction. Scale bars in micrometres [μm]. All PCM. B, E, F, H–*Macrobiotus harmsworthi obscurus* ssp. nov. (Dastych 1985) (= *Mesobiotus harmsworthi* s.s. (Murray, 1907)–photos of paratypes from ZMHU.

**Fig 4:**
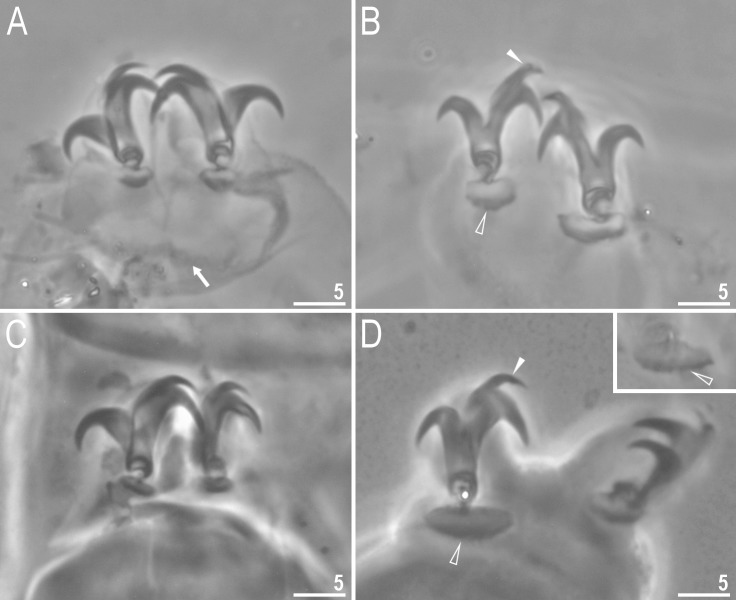
*Mesobiotus harmsworthi*–claws. A, C–claws III and II respectively with smooth lunules; arrow indicates cuticular bar under claws; B, D–claws IV with indented lunules (empty arrowheads) (insert–well visible indented lunules); the filled arrowheads indicate large accessory points on claws IV. Scale bars in micrometres [μm]. All PCM. C–D–*Macrobiotus harmsworthi obscurus* ssp. nov. (Dastych 1985) (= *Mesobiotus harmsworthi* s.s. (Murray, 1907)–photos of paratypes from ZMHU.

**Fig 5:**
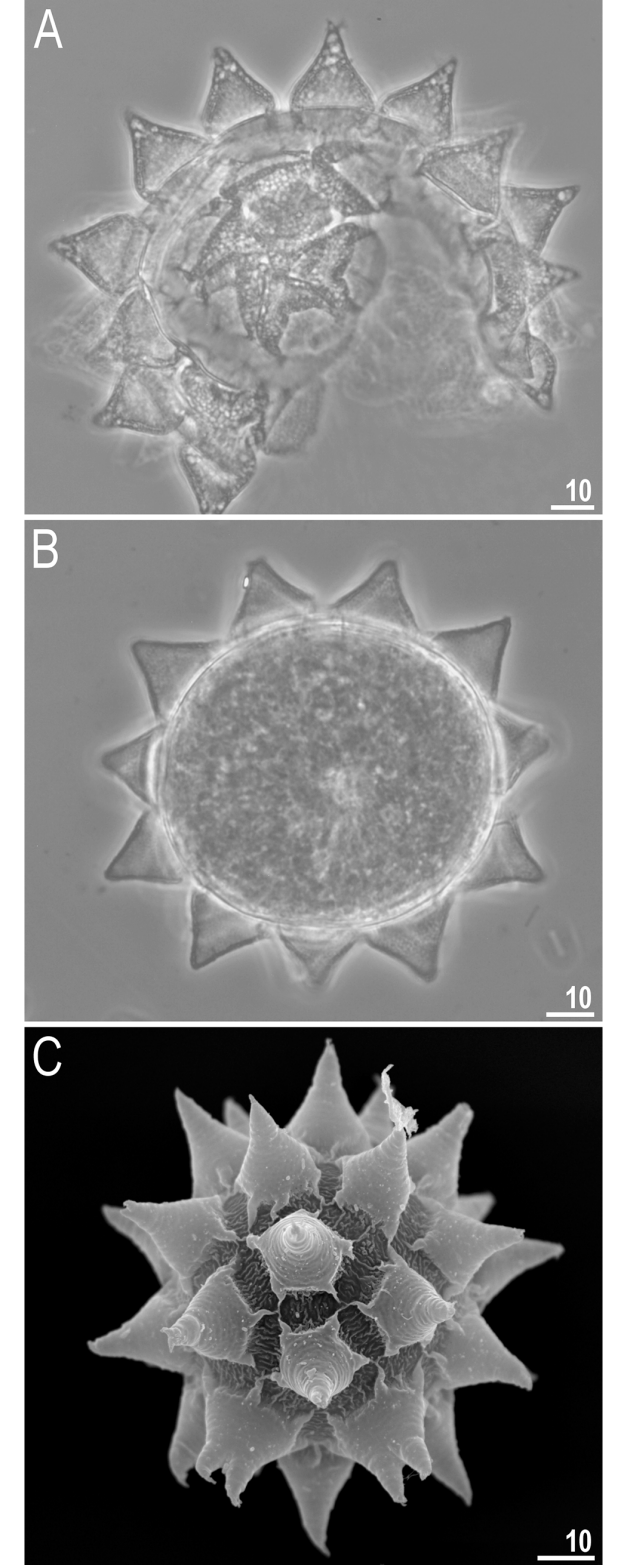
*Mesobiotus harmsworthi*–eggs. A–egg chorion visible in PCM; B–egg midsection visible in PCM; C–egg chorion visible in SEM. Scale bars in micrometres [μm].

**Fig 6:**
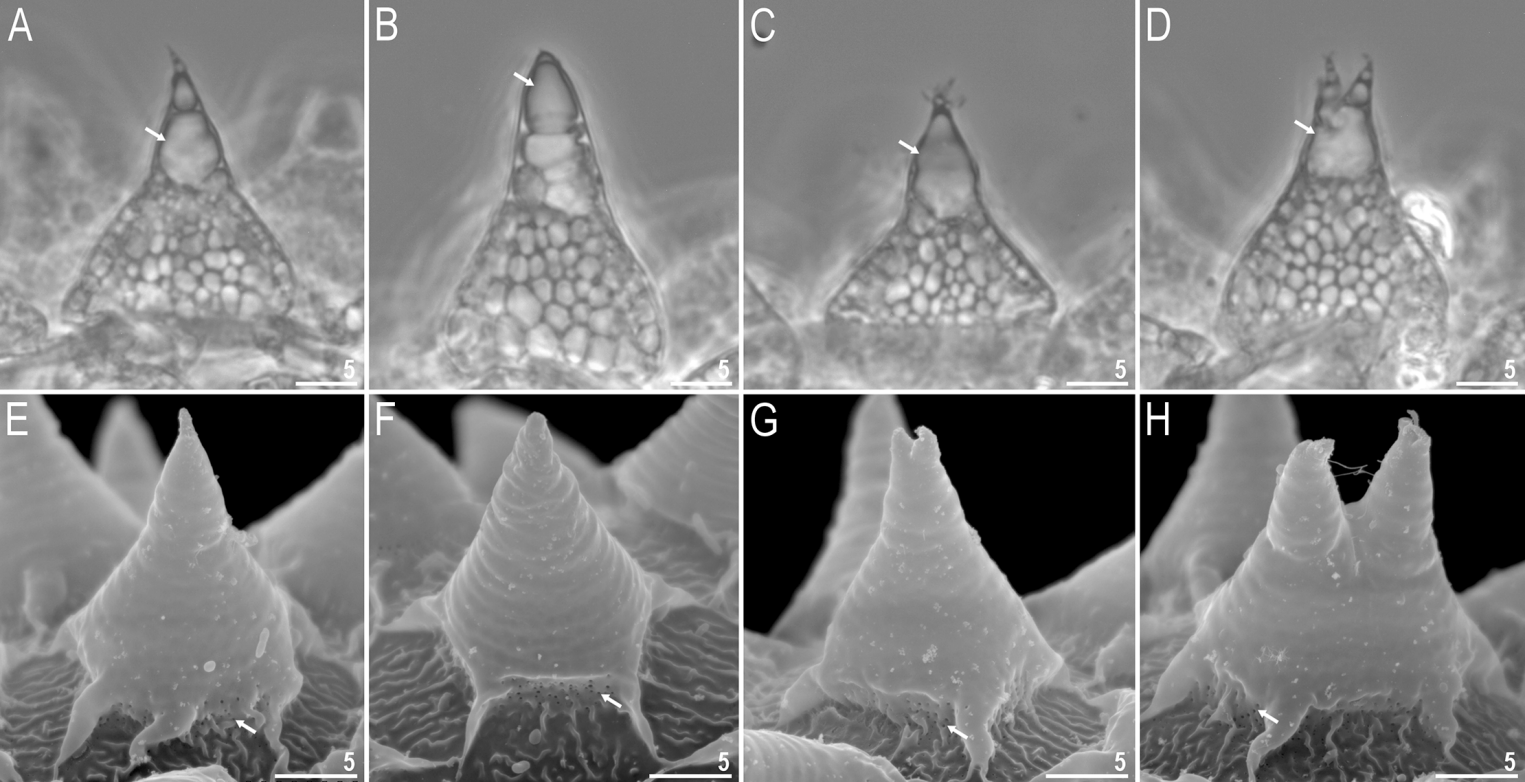
*Mesobiotus harmsworthi*–egg processes morphology. A–D–egg process morphology seen in PCM; arrows indicate a single, large bubble in the apex; E–H–egg process morphology seen in SEM; arrows indicate small pores at process bases. Scale bars in micrometres [μm].

**Fig 7:**
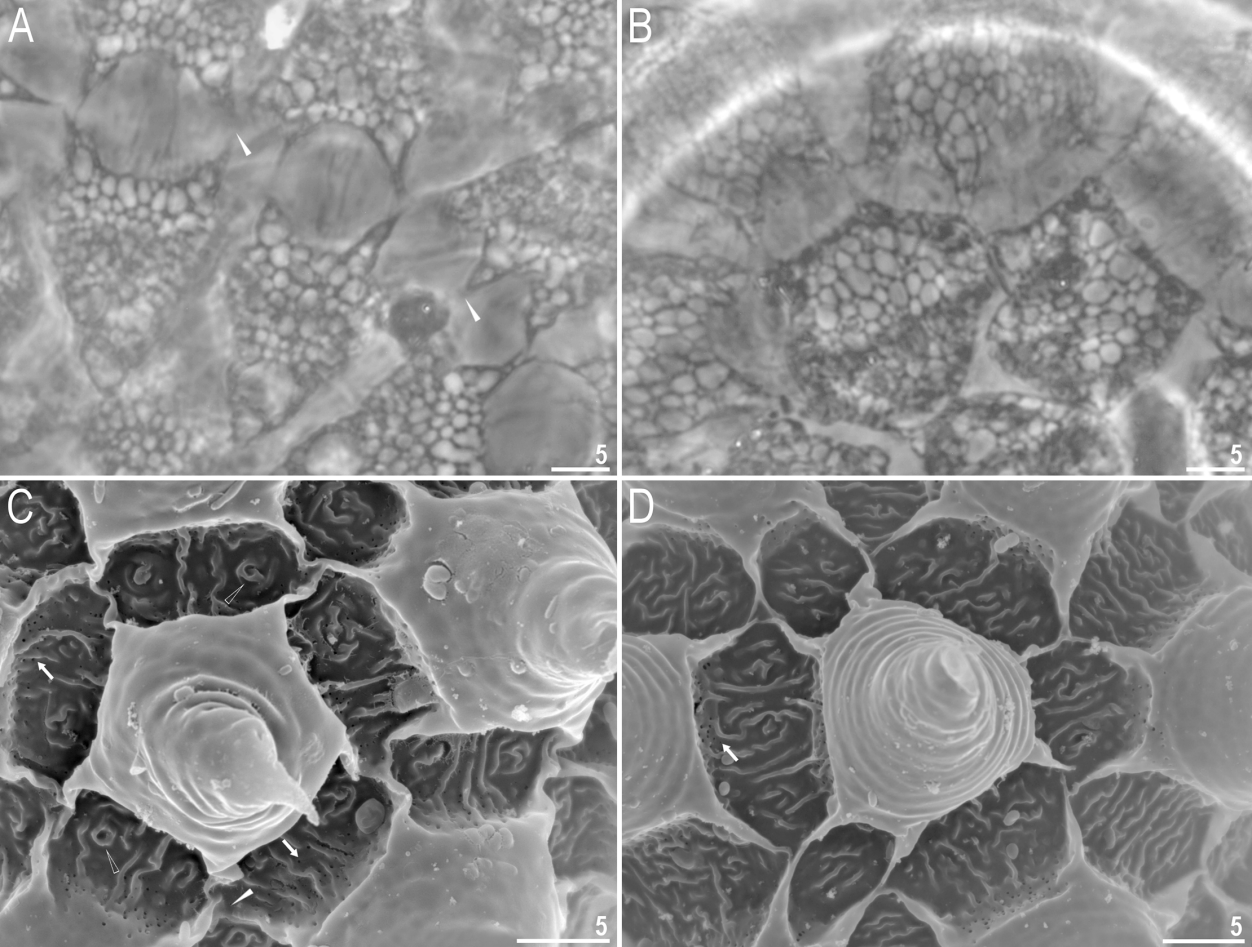
*Mesobiotus harmsworthi*–egg chorion. A–B–the surface between egg processes visible in PCM; filled arrowheads indicate not fully closed areoles; C–D–the surface between egg processes visible in SEM; filled arrowhead indicate not fully closed areoles; empty arrowheads indicate whirls inside areolae; arrows indicate small pores at process bases. Scale bars in micrometres [μm].

Macrobiotus harmsworthi obscurus Dastych, 1985 [64]

(Figs 2–7 and 18; Tables 4 and 5)

**Table 4:** Measurements and *pt* values of selected morphological structures of the specimens of paratypes of *Macrobiotus harmsworthi obscurus* ssp. nov. (Dastych 1985) (= *Mesobiotus harmsworthi s*.*s*. (Murray, 1907) (N—number of specimens/structures measured, RANGE refers to the smallest and the largest structure among all measured specimens; SD—standard deviation).

CHARACTER	N	RANGE						MEAN		SD	
		μm			*pt*			μm	*pt*	μm	*pt*
Body length	11	275	–	489		*–*		373		54	
Buccal tube							* *		* *		* *
Buccal tube length	23	37.3	–	62.0		–	* *	43.5	*–*	5.9	*–*
Stylet support insertion point	23	28.9	–	49.2	*77*.*1*	*–*	*80*.*0*	34.0	*78*.*2*	4.8	*0*.*8*
Buccal tube external width	23	4.9	–	7.5	*12*.*1*	*–*	*16*.*4*	6.3	*14*.*5*	0.7	*1*.*0*
Buccal tube internal width	23	3.4	–	5.8	*9*.*1*	*–*	*11*.*4*	4.5	*10*.*3*	0.6	*0*.*7*
Ventral lamina length	23	24.0	–	40.5	*62*.*3*	*–*	*66*.*8*	28.2	*64*.*8*	3.8	*1*.*4*
Placoid lengths							* *		* *		* *
Macroplacoid 1	23	4.6	–	7.9	*12*.*3*	*–*	*16*.*7*	6.5	*14*.*8*	1.0	*1*.*2*
Macroplacoid 2	23	3.8	–	6.7	*10*.*0*	*–*	*13*.*3*	5.1	*11*.*7*	0.7	*1*.*0*
Macroplacoid 3	23	4.5	–	7.6	*11*.*9*	*–*	*15*.*5*	6.0	*13*.*6*	1.0	*1*.*1*
Microplacoid	22	2.1	–	3.5	*5*.*6*	*–*	*7*.*9*	3.0	*6*.*8*	0.4	*0*.*6*
Macroplacoid row	23	15.5	–	25.4	*41*.*0*	*–*	*49*.*9*	19.8	*45*.*6*	2.7	*2*.*4*
Placoid row	23	18.7	–	30.5	*49*.*2*	*–*	*59*.*5*	23.8	*54*.*7*	3.2	*2*.*8*
Claw 1 lengths							* *		* *		* *
External primary branch	15	8.5	–	11.8	*18*.*7*	*–*	*26*.*1*	9.8	*22*.*1*	1.1	*1*.*9*
External secondary branch	8	6.5	–	9.9	*16*.*0*	*–*	*21*.*5*	8.2	*18*.*3*	1.3	*1*.*8*
Internal primary branch	14	7.6	–	10.8	*16*.*5*	*–*	*22*.*6*	9.2	*20*.*6*	0.9	*1*.*6*
Internal secondary branch	4	5.8	–	8.7	*15*.*4*	*–*	*18*.*6*	7.6	*17*.*0*	1.3	*1*.*8*
Claw 2 lengths							* *		* *		* *
External primary branch	15	8.5	–	12.6	*19*.*9*	*–*	*26*.*1*	10.0	*23*.*4*	1.2	*1*.*8*
External secondary branch	11	7.1	–	10.5	*17*.*8*	*–*	*23*.*2*	8.4	*19*.*9*	1.1	*1*.*7*
Internal primary branch	11	7.9	–	11.0	*19*.*3*	*–*	*22*.*9*	9.1	*21*.*2*	0.9	*1*.*1*
Internal secondary branch	7	6.9	–	9.4	*16*.*4*	*–*	*19*.*1*	7.6	*18*.*1*	0.8	*1*.*1*
Claw 3 lengths							* *		* *		* *
External primary branch	15	8.4	–	12.6	*19*.*7*	*–*	*27*.*9*	10.5	*23*.*5*	1.5	*2*.*0*
External secondary branch	11	7.1	–	11.0	*14*.*8*	*–*	*23*.*7*	8.6	*19*.*7*	1.1	*2*.*4*
Internal primary branch	17	7.9	–	11.6	*18*.*2*	*–*	*25*.*7*	9.4	*21*.*6*	1.1	*1*.*8*
Internal secondary branch	6	6.8	–	8.7	*13*.*2*	*–*	*19*.*2*	7.6	*17*.*1*	0.7	*2*.*0*
Claw 4 lengths							* *		* *		* *
Anterior primary branch	16	9.4	–	14.0	*23*.*3*	*–*	*29*.*6*	11.4	*26*.*2*	1.3	*1*.*5*
Anterior secondary branch	11	7.4	–	10.3	*19*.*2*	*–*	*21*.*6*	8.8	*20*.*1*	0.8	*0*.*7*
Posterior primary branch	17	10.5	–	15.4	*26*.*6*	*–*	*33*.*4*	12.5	*29*.*1*	1.6	*1*.*7*
Posterior secondary branch	13	8.0	–	11.1	*20*.*3*	*–*	*23*.*9*	9.3	*21*.*7*	1.1	*1*.*0*

**Table 5:** Measurements [in μm] of selected morphological structures of the eggs of *Macrobiotus harmsworthi obscurus* ssp. nov. (Dastych 1985) (= *Mesobiotus harmsworthi s*.*s*. (Murray, 1907) (N—number of specimens/structures measured, RANGE refers to the smallest and the largest structure among all measured eggs; SD—standard deviation).

CHARACTER	N	RANGE			MEAN	SD
Egg bare diameter	9	71.2	–	84.6	77.9	3.6
Egg full diameter	9	101.0	–	120.0	108.5	5.9
Process height	27	14.3	–	19.7	16.7	1.2
Process base width	27	15.2	–	21.4	18.5	1.4
Process base/height ratio	27	103%	–	125%	110%	5%
Distance between processes	27	3.4	–	7.0	5.5	0.9
Number of processes on the egg circumference	9	11	–	12	11.3	0.5

### Material examined

**Type material (original slide labelling):** 50 paratypes (41 animals and 9 eggs) of *Macrobiotus harmsworthi obscurus* ssp. nov. [15]): (1) Spitsb. 150, Vest Spitsbergen, Störmerfjellet Mt, 1295m, Sept. 1977, lg. H. Dastych, Eing.Nr.A71/93 (16 animals); (2) Spitsb. 152, Vest Spitsbergen, Störmerfjellet Mt, 1295m, Sept. 1977, lg. H. Dastych, Eing.Nr.A71/93 (23a and 7e); (3) Spits. 153, Vest Spitsbergen, Störmerfjellet Mt, 1295m, Sept. 1977, lg. H. Dastych, Eing.Nr.A71/93 (1a and 1e); (4) Spits. 154, Vest Spitsbergen, Störmerfjellet Mt, 1295m, Sept. 1977, lg. H. Dastych, Eing.Nr.A71/93 (1a and 1e). At present these specimens should be considered as the neotype series.

**Additional material: I) Spitsbergen, Hornsund, Revdalen**: **1)** 77°01'39''N, 15°22'47''E, 76 m asl, moss on rock, northern part of the Revdalen, near the Revvatnet and the Revelva (4 animals and 2 eggs); **2)** 77°01'34''N, 15°23'12''E, 76 m asl, moss on rock, northern part of the Revdalen, near the Revvatnet and the Revelva (4a and 9e); **3)** 77°01'09''N, 15°24'34''E, 50 m asl, moss on rock, northern part of the Revdalen, near the Revvatnet (southern edge) and the Revelva (9a and 1e) [65]; **II) Spitsbergen, Hornsund, Ariekammen: 1)** 77°01'10''N, 15°31'16''E, 524 m asl, moss on rock (16a and 6e); **2)** 77°01'04''N, 15°31'11''E, 450 m asl, moss on rock (5a and 1e); **3)** 77°00'58''N, 15°31'10''E, 400 m asl, moss on rock (1a); **4)** 77°00'48''N, 15°30'58''E, 350 m asl, moss on rock, (2a), **5)** 77°00'47''N, 15°31'12''E, 300 m asl, lichen on rock, moss on rock (30a and 12e), **6)** 77°00'43''N, 15°31'16''E, 250 m asl, moss on rock (4a and 5e); **7)** 77°00'36''N, 15°31'10''E, 150 m asl, moss on rock, (9a and 1e), **8)** 77°00'31''N, 15°31'43''E, 50 m asl, moss on rock, (6a and 8e) [66]; **9)** *ca*. 77°0'26.4"N, 15°32'43.02"E, 9 m asl, moss on soil (4a and 2e); **10)** *ca*. 77°0'35.1"N, 15°32'0.3"E, 28 m asl, moss on soil (1a) [67]. **III) Phippsøya: 1)** 80°41.211’N; 20°50.606’E, 47 m asl, moss on rock (9a and 10e).

### Redescription of *Mesobiotus harmsworthi*

#### Animals (morphometrics in Table 4)

Body white in living specimens and transparent after fixation (Fig 2A and 2B). Eyes present. Cuticle smooth, *i*.*e*., without gibbosities, papillae, spines, sculpture or pores. Granulation present only on the external surface of all legs (Fig 2C–2F).

Bucco-pharyngeal apparatus of the *Macrobiotus* type (Fig 3A and 3B), with the ventral lamina and ten peribuccal lamellae. Mouth antero-ventral. The oral cavity armature well developed and composed of three bands of teeth (Fig 3C–3F). The first band of teeth is composed of numerous small granules arranged in a several rows situated anteriorly in the oral cavity, just behind the bases of the peribuccal lamellae (Fig 3C and 3F; arrowhead). The band is hardly detectible under PCM in small specimens and clearly visible in large individuals. The second band of teeth is situated between the ring fold and the third band of teeth and comprises ridges parallel to the main axis of the buccal tube and additional teeth between and below them, larger than those in the first band (Fig 3C, 3E and 3F; arrow). The teeth of the third band are located within the posterior portion of the oral cavity, between the second band of teeth and the buccal tube opening (Fig 3C–3F; indented arrowhead). The third band of teeth is divided into the dorsal and the ventral portion. Under PCM, both dorsal and ventral teeth are visible as two lateral and one median transverse ridges (Fig 3C–3F; indented arrowhead). Pharyngeal bulb spherical, with triangular apophyses, three rod-shaped macroplacoids and a triangular microplacoid. Macroplacoid length sequence 2<3≤1. The first macroplacoid narrower anteriorly, the second without constrictions and the third with a small, subterminal constriction (Fig 3G and 3H; empty arrowhead).

Claws of the *Mesobiotus* type, robust (Fig 4A–4D). Primary branches with distinct accessory points. Accessory point on claws IV are larger and more protruding than in most macrobiotids (Fig 4B and 4D; arrowheads). Lunules under claws I–III smooth and slightly dentated under claws IV (Fig 4B and 4D; empty arrowhead). Thin cuticular bars under claws I–III present (Fig 4A, arrow). Other cuticular structures on legs absent.

#### Eggs (morphometrics in Table 5)

Laid freely, white, spherical and ornamented, with processes and delicate areolation (Figs 5–7). Egg processes in the shape of wide cones (Fig 6A–6H). The cones can be slightly concave (Figs 5B, 5C, 6B and 6C) or sigmoidal, *i*.*e*., with a slightly swollen base and a narrowed apex (Fig 6D). The processes with a single sharp (Fig 6A and 6E) or slightly blunt (Fig 6B and 6F) apex, only occasionally bifurcated (Fig 6D, 6G and 6H). In PCM, processes reticulated with mesh size 0.5–2.0 μm in diameter, evidently larger near the process base and apex (Fig 6A–6D). Sometimes, instead of several large meshes, a single very large bubble is present in the apex (Fig 6A–6D, arrows). In SEM, processes smooth, but with well visible small pores at the bases and inside the areoles close to the processes (Figs 6E–6H, 7C and 7D, arrows). Each process surrounded by five or six areolae delimited by thin brims (Fig 7A–7D). The brims are very often discontinuous, thus areolae are not always fully formed (Fig 7A and 7C, arrowheads). Surface inside the areolae with clearly visible wrinkles, both in PCM (Fig 7A and 7B) and in SEM (Fig 7C and 7D). Occasionally, the wrinkles may form a small whirl in the areola centre (Fig 7C, empty arrowhead).

#### DNA sequences

We obtained sequences for all four analysed genetic markers from the three sequenced syngenophores collected from Spitsbergen and an additional COI sequence from a syngenophore collected from Phippsøya (see Table 1 for details). All nuclear markers were represented by a single haplotypes whereas COI exhibited two distinct haplotypes (separated by the p-distance of 0.7%):

The **18S rRNA** sequence (GenBank: MH197146), 829 bp long:

The **28S rRNA** sequence (GenBank: MH197264), 751 bp long:

The **ITS-2** sequence (GenBank: MH197154), 415 bp long:

The **COI** haplotype 1 sequence (GenBank: MH195150), 621 bp long:

The **COI** haplotype 2 sequence (GenBank: MH195151), 621 bp long:

#### Etymology

Although Murray [11] did not explain the choice of the species name, it seems reasonable to assume that *M*. *harmsworthi* was named after Cape Mary Harmsworth in Franz Joseph Land, which is one of the type localities mentioned in the original description of the species.

#### Neotype locality

Norway; 79°2′25″N, 16°42′12″E, 1,295 m asl, Svalbard, Spitsbergen, Størmerfjellet Mt.

#### Distribution

Norway; Svalbard Archipelago: Spitsbergen (Albert I Land, Andrée Land, Atomfjella, Bünsow Land and Hornsund), Phippsøya; Russia; Franz Josef Land, Perm Krai; United Kingdom: Shetland Islands [11, 15, 64].

#### Remarks

In our opinion the presence of this species in Perm Krai needs to be confirmed, especially in the light of the description of a new species *Mesobiotus skorackii* **sp. nov.** from the Kyrgyz Republic. Specimens designated as paratypes by Dastych [15] were collected in few localities. According to International Commission on Zoological Nomenclature type specimens should be collected from the same locality. However, since all specimens appear to represent a single species, we decided not to change original designations of type specimens.

#### Phenotypic differential diagnosis

*Mesobiotus harmsworthi*, by the presence of smooth cuticle and egg areolation (although not always fully developed), is most similar to *M*. *barbarae*, *M*. *ethiopicus*, *M*. *hieronimi* (Pilato & Claxton, 1988 [27]), *M*. *nuragicus* (Pilato & Sperlinga, 1975 [26]), *M*. *ovostriatus* (Pilato & Patanè, 1998 [28]), *M*. *peterseni* (Maucci, 1991 [29]), *M*. *pseudoliviae* (Pilato & Binda, 1996 [31]) and *M*. *skorackii* **sp. nov.**, but differs from all of them by large and protruding accessory points on claws IV. Additionally, *M*. *harmsworthi* differs specifically from:

1. ***M*. *barbarae*** (known only from type locality in the Dominican Republic [32]) by: the presence of additional teeth in the second band of teeth, an undivided ventro-median tooth in the third band of teeth, the presence of granulation on legs I, a different macroplacoid length sequence (2<3≤1 in *M*. *harmsworthi vs* 2<1<3 in *M*. *barbarae*), a different morphology of egg process apex (apices not defined in *M*. *harmsworthi vs* processes terminated by a thin, flexible apex in *M*. *barbarae*), and by the presence of evidently larger meshes near the bases of egg processes (uniform mesh size in *M*. *barbarae*) and by the areoles not always fully formed on the egg surface.
2. ***M*. *ethiopicus*** (known only from type locality in Ethiopia [34]) by: the presence of eyes, the absence of evidently larger teeth in the second band of teeth, the presence of additional teeth in the oral cavity situated between second and third band of teeth and by a different morphology of egg process apex (a single, occasionally bifurcated apex in *M*. *harmsworthi vs* processes terminated by several short, thin, and flexible filaments susceptible to fracture in *M*. *ethiopicus*).
3. ***M*. *hieronimi*** (known only from Australia and probably South Georgia [27]) by: the never joined teeth in second band of teeth, the presence of dentated lunules under claws IV, stylet supports in a more posterior position (*pt = 77*.*1–80*.*0* in *M*. *harmsworthi vs pt = 73*.*3–74*.*8* in *M*. *hieronimi*), by the areoles not always fully formed on the egg surface and by evidently larger meshes near the bases of the egg processes (uniform mesh size in *M*. *hieronimi*).
4. ***M*. *nuragicus*** (known from Europe, Africa, Indonesia, South and Central America [5, 68, 69, 70]) by: the presence of additional teeth in second band of teeth, a different macroplacoid length sequence (2<3≤1 in *M*. *harmsworthi vs* 2<1<3 in *M*. *nuragicus*), a different morphology of egg process apex (apices only occasionally bifurcated in *M*. *harmsworthi vs* apices always divided into several short filaments in *M*. *nuragicus*), the presence of larger meshes near the bases and apices of egg processes (uniform mesh size in *M*. *nuragicus* and by the areoles not always fully formed on the egg surface.
5. ***M*. *ovostriatus*** (known only from type locality in Argentina [28]) by: the presence of the first band of teeth (in PCM), a better developed second band of teeth (second band of teeth reduced and composed of small granular teeth in *M*. *ovostriatus*), slightly dentated lunules under claws IV (smooth in *M*. *ovostriatus*), evidently larger meshes near the bases and apices of egg processes (uniform mesh size in *M*. *ovostriatus*), a different morphology of egg process apex (a single, occasionally bifurcated apex in *M*. *harmsworthi vs* processes terminated by a thin, flexible apex in *M*. *ovostriatus*) and by the areoles not always fully formed on the egg surface.
6. ***M*. *peterseni*** (known only from type locality in Greenland [29]): a different macroplacoid length sequence (2<3≤1 in *M*. *harmsworthi vs* 2<1<3 in *M*. *peterseni*) and a different shape of egg processes (sharp cones in the *M*. *harmsworthi vs* blunt domes in *M*. *peterseni*).
7. ***M*. *pseudoliviae*** (known only from type locality in New Zealand [31]) by: the presence of additional teeth in second band of teeth, the teeth in second band never joined, evidently larger meshes near the bases and apices of egg processes (uniform mesh size in *M*. *pseudoliviae*), the areoles not always fully formed on the egg surface, fewer areoles around the egg processes (6 in *M*. *harmsworthi vs ca*. 16 in *M*. *pseudoliviae*), a smaller egg full diameter (101.0–120.0 μm in *M*. *harmsworthi vs* 156.0–177.0 μm in *M*. *pseudoliviae*), shorter egg processes (14.3–19.7 μm in *M*. *harmsworthi vs* 42.0–56.0 μm in *M*. *pseudoliviae*), and by narrower process bases (15.2–21.4 μm in *M*. *harmsworthi vs* 28.0–45.0 μm in *M*. *pseudoliviae*). Maximum dimensions (both absolute and relative) of all morphological structures are smaller in *M*. *harmsworthi*, but the morphometric comparison of the two species must be treated with caution because only one specimen was measured in the original description of *M*. *pseudoliviae*.
8. ***M*. *skorackii* sp. nov.**–please see the description and the differential diagnosis below.

#### Genotypic differential diagnosis

The ranges of uncorrected genetic p-distances between the new species and species of the genus *Mesobiotus*, for which sequences are available from GenBank, were as follows:

**18S rRNA**: 0.4–5.8% (3.7% on average), with the most similar being *M*. *occultatus* from Spitsbergen (MH197147) and the least similar being *M*. cf. *mottai* and *M*. *furciger* from the Antarctic (KT226072 and EU266928, respectively) and an undetermined *M*. *furciger* group species from Norway (MH197148);

**28S rRNA**: 3.2–9.2% (7.0% on average), with the most similar being an undetermined *M*. *harmsworthi* group species from Russia (MH197266) and the least similar being *M*. *radiatus* Pilato, Binda & Catanzaro, 1991 [71] from Kenya (MH197152);

**ITS-2**: 10.3–29.4% (19.4% on average), with the most similar being *M*. *occultatus* from Spitsbergen (MH197155) and the least similar being an undetermined *M*. *furciger* group species from Norway (MH197156);

**COI**: 17.5–23.5% (21.4% on average), with the most similar being an undetermined *M*. *furciger* group species from Norway (MH195153) and the least similar “*M*. *harmsworthi*” from China (GU113140).

### *Mesobiotus occultatus* sp. nov

urn:lsid:zoobank.org:act:0C31B770-CA14-4664-B923-160737EF0618

*Macrobiotus harmsworthi harmsworthi* Murray, 1907 [65]

*M*. *harmsworthi harmsworthi* Murray, 1907 [66, 67]

**Fig 8:**
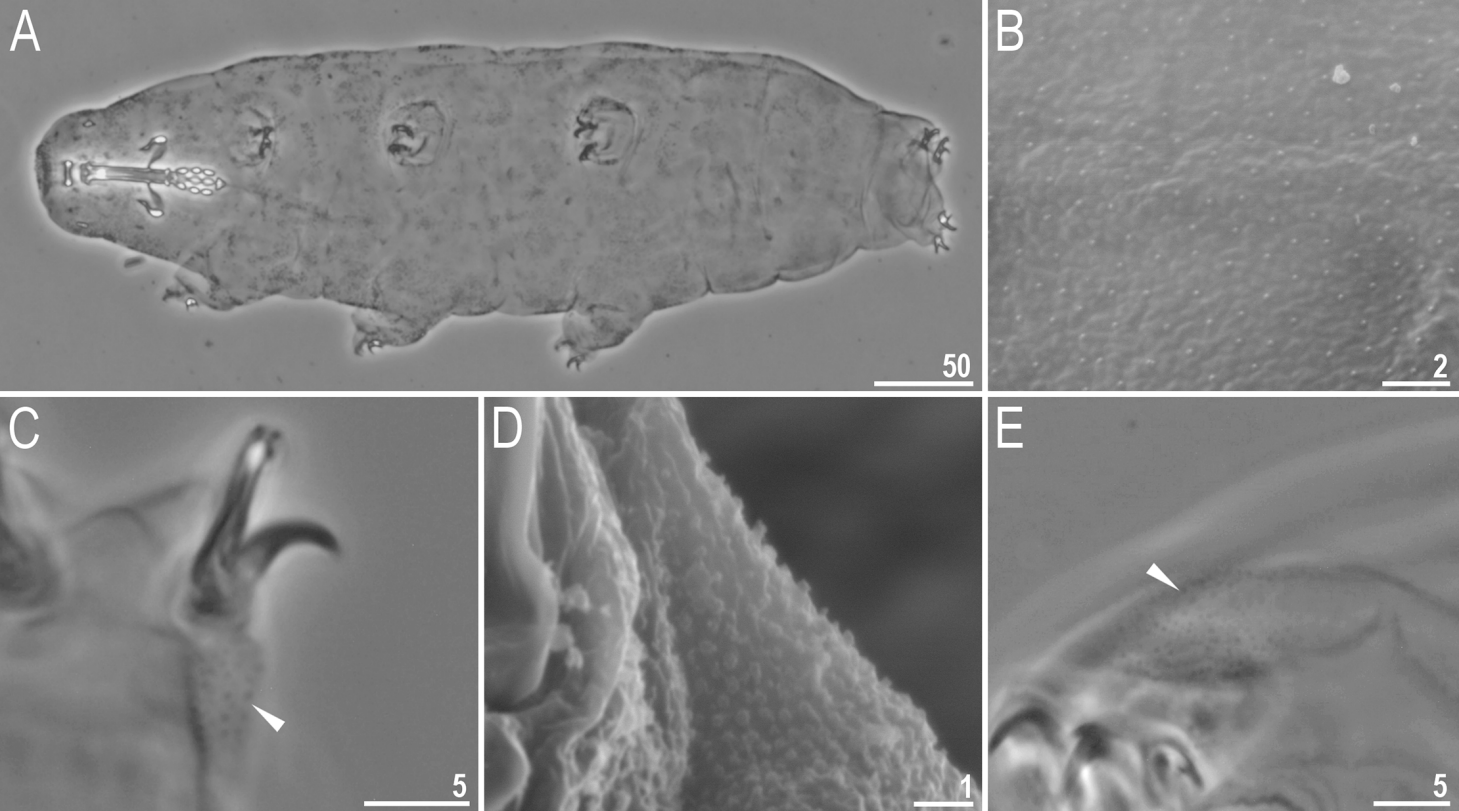
*Mesobiotus occultatus* sp. nov.–habitus and granulation on legs. A–dorso-ventral projection of the entire animal (holotype, PCM); B–body microgranulation visible in SEM (paratype); C–granulation on leg II, arrowhead (paratype, PCM); D–granulation on leg III (paratype, SEM); E–granulation on leg IV, arrowhead (paratype, PCM). Scale bars in micrometres [μm].

**Fig 9:**
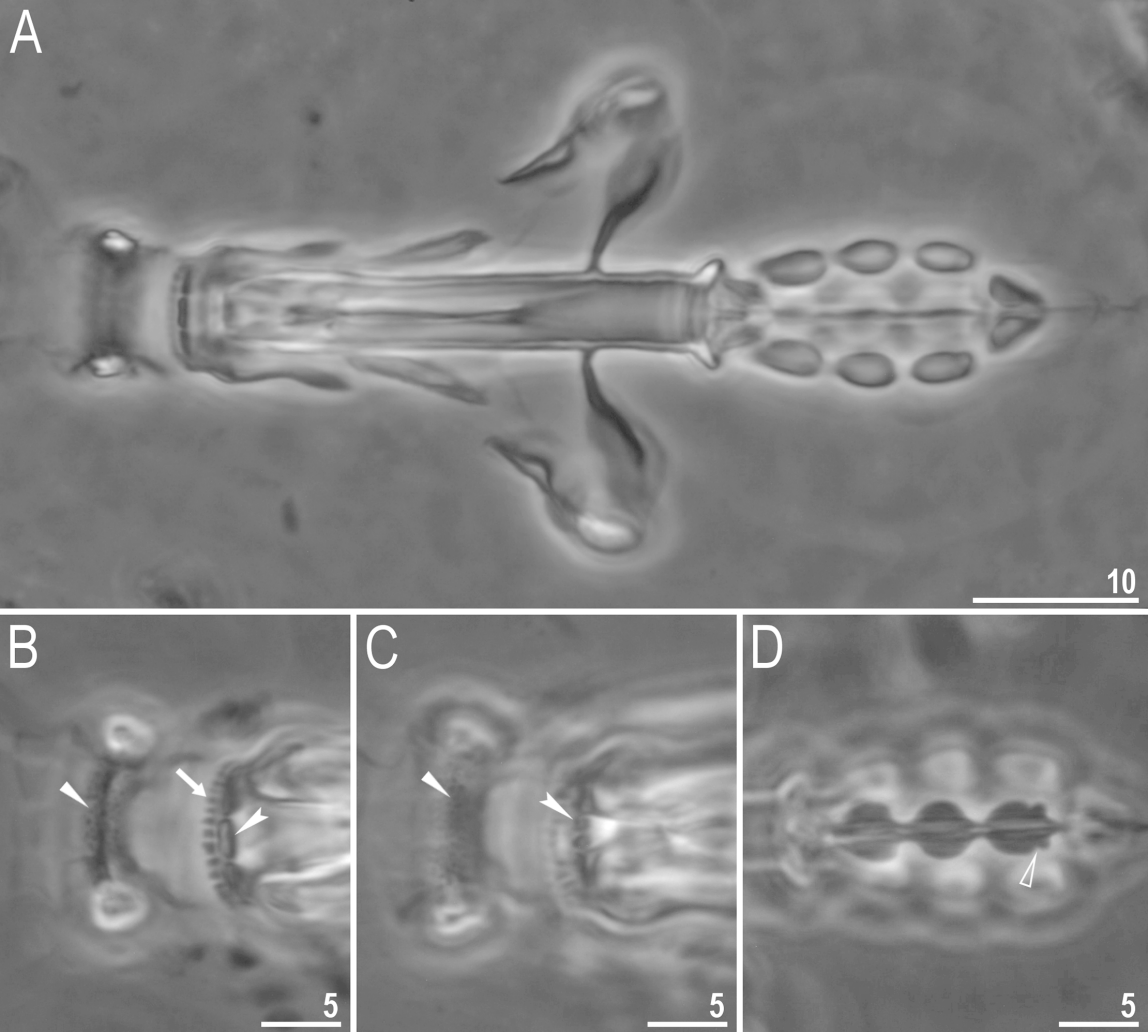
*Mesobiotus occultatus* sp. nov.–buccal apparatus and the oral cavity armature. A–general view (paratype); B–C–oral cavity armature; filled flat arrowheads indicate teeth of the first band, the arrow indicates teeth of the second band, indented arrowheads indicate teeth of the third band (paratype); D–ventral placoids; the empty arrowhead indicates the subterminal constriction in the third macroplacoid (paratype). Scale bars in micrometres [μm]. All PCM.

**Fig 10:**
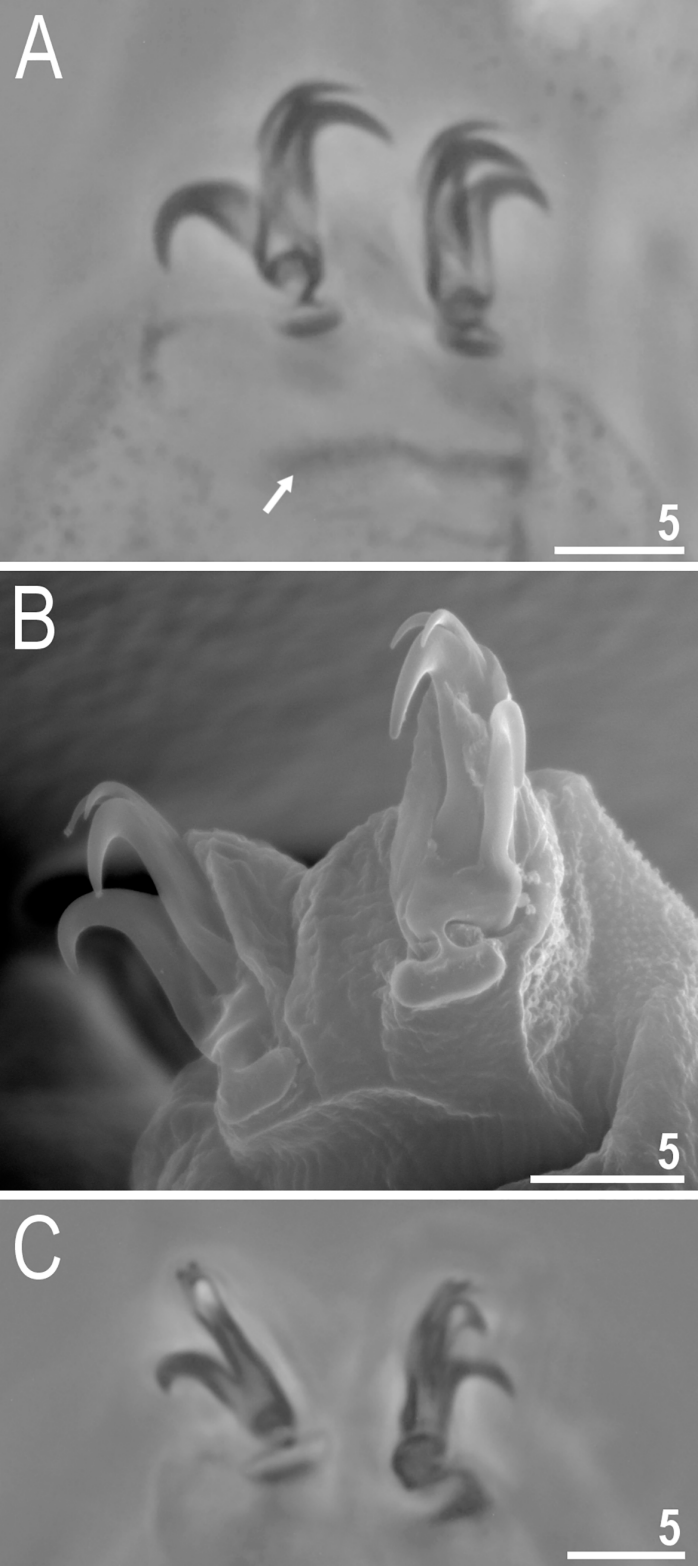
*Mesobiotus occultatus* sp. nov.–claws. A–claws I with smooth lunules; arrow indicates the cuticular bar under claws (holotype, PCM); B–claws III with smooth lunules (paratype, SEM); C–claws IV with smooth lunules (paratype, PCM). Scale bars in micrometres [μm].

**Fig 11:**
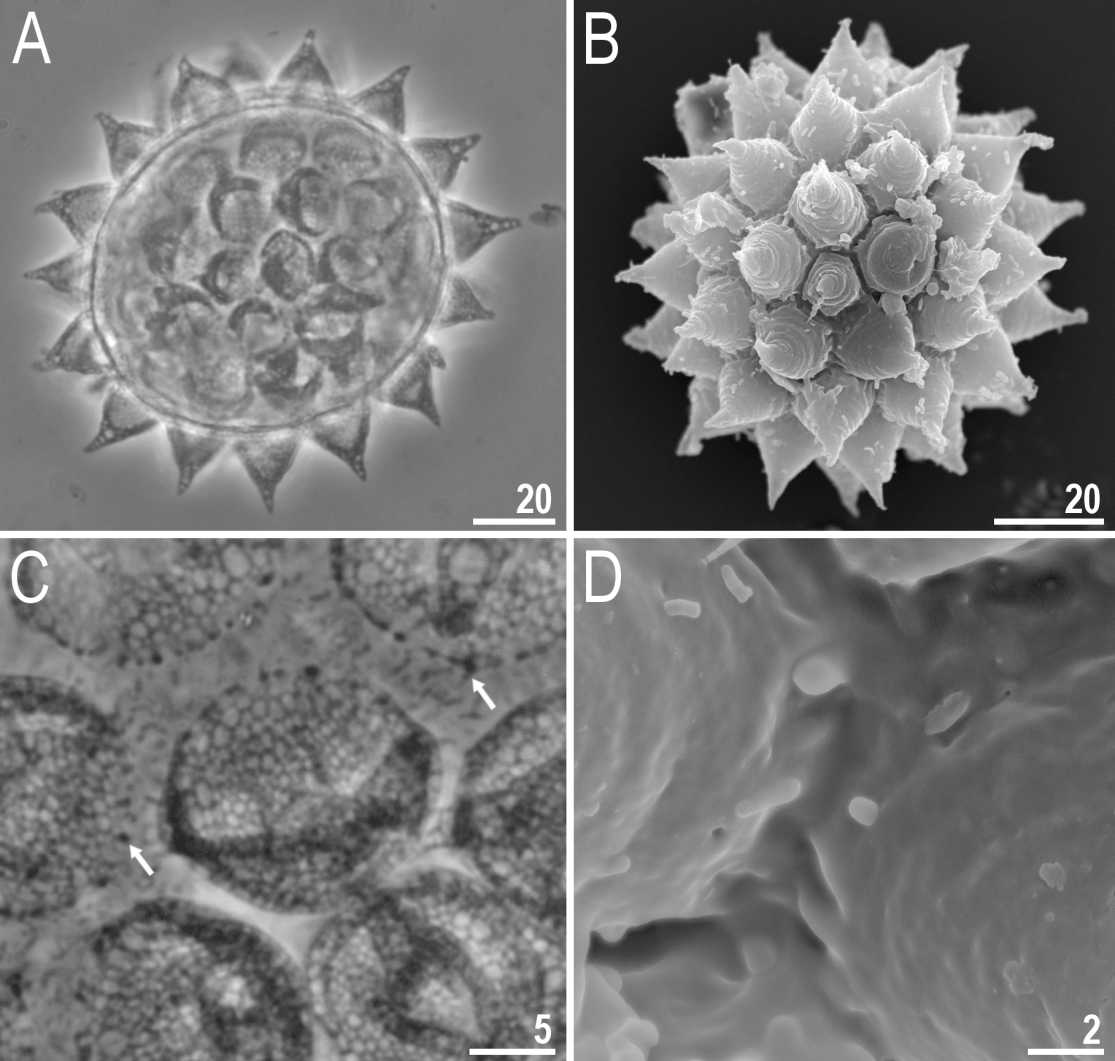
*Mesobiotus occultatus* sp. nov.–eggs. A–B–egg chorion visible in PCM and SEM respectively; C–the surface between egg processes visible in PCM; arrows indicate a crown of small thickenings at the base of processes (PCM); D–the surface between egg processes visible in SEM. Scale bars in micrometres [μm].

**Fig 12:**
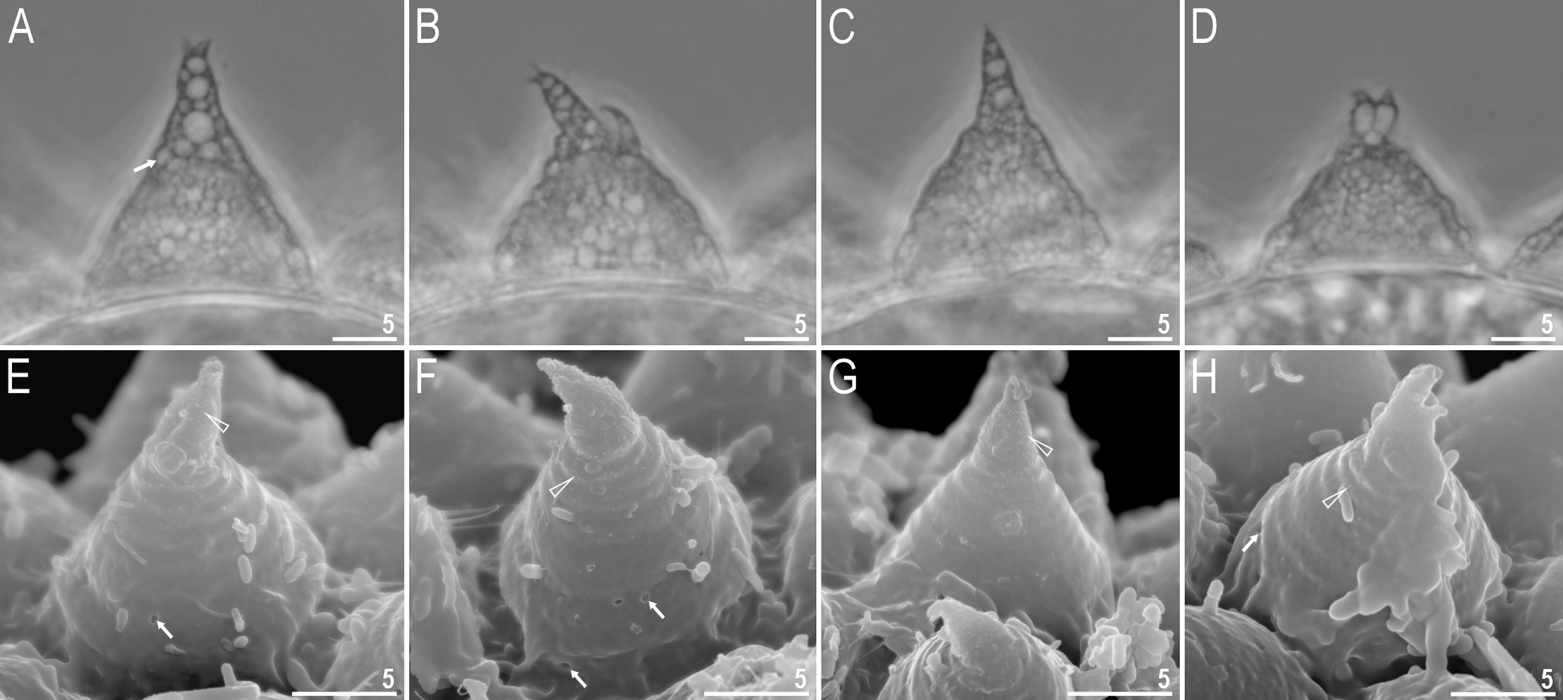
*Mesobiotus occultatus* sp. nov.–egg processes morphology. A–D–egg process morphology seen in PCM; E–H–egg process morphology seen in SEM; arrows indicate small pores at the process bases, empty arrowheads indicate fine granulation present at the apical part of the processes. Scale bars in micrometres [μm].

*Mesobiotus harmsworthi harmsworthi* (Murray, 1907) [72]

(Figs 8–12; Tables 6 and 7)

**Table 6:** Measurements and *pt* values of selected morphological structures of *Mesobiotus occultatus* sp. nov. mounted in Hoyer’s medium (N—number of specimens/structures measured, RANGE refers to the smallest and the largest structure among all measured specimens; SD—standard deviation).

CHARACTER	N	RANGE						MEAN		SD		Holotype	
		μm			*pt*			μm	*pt*	μm	*pt*	μm	*pt*
Body length	14	244	–	535		*–*		345		76		353	
Buccal tube							* *		* *		* *		
Buccal tube length	14	27.4	–	47.9		–	* *	37.4	*–*	6.3	*–*	37.2	*–*
Stylet support insertion point	14	20.9	–	37.1	*75*.*5*	*–*	*78*.*7*	28.9	*77*.*3*	5.0	*0*.*8*	28.7	*77*.*2*
Buccal tube external width	14	4.0	–	7.5	*13*.*7*	*–*	*15*.*7*	5.5	*14*.*7*	1.0	*0*.*7*	5.6	*15*.*1*
Buccal tube internal width	14	2.5	–	5.4	*8*.*8*	*–*	*11*.*3*	3.7	*9*.*9*	0.8	*0*.*8*	3.9	*10*.*5*
Ventral lamina length	14	17.0	–	29.8	*59*.*3*	*–*	*64*.*9*	23.4	*62*.*5*	3.9	*1*.*5*	22.8	*61*.*3*
Placoid lengths							* *		* *		* *		
Macroplacoid 1	14	3.2	–	7.1	*11*.*7*	*–*	*14*.*8*	5.0	*13*.*1*	1.2	*1*.*1*	4.8	*12*.*9*
Macroplacoid 2	14	2.7	–	6.0	*9*.*7*	*–*	*12*.*6*	4.2	*11*.*2*	1.0	*1*.*0*	4.4	*11*.*8*
Macroplacoid 3	14	2.9	–	6.7	*10*.*6*	*–*	*14*.*0*	4.7	*12*.*4*	1.1	*0*.*9*	4.6	*12*.*4*
Microplacoid	14	2.4	–	5.5	*8*.*0*	*–*	*12*.*1*	3.8	*10*.*1*	1.0	*1*.*3*	4.2	*11*.*3*
Macroplacoid row	14	10.4	–	22.7	*37*.*5*	*–*	*47*.*4*	15.9	*42*.*1*	3.6	*2*.*8*	15.4	*41*.*4*
Placoid row	14	13.3	–	29.9	*48*.*5*	*–*	*62*.*4*	20.8	*55*.*0*	4.8	*4*.*0*	20.5	*55*.*1*
Claw 1 lengths							* *		* *		* *		
External primary branch	11	7.2	–	12.1	*23*.*4*	*–*	*27*.*4*	9.0	*24*.*7*	1.4	*1*.*3*	8.7	*23*.*4*
External secondary branch	10	5.1	–	10.6	*18*.*6*	*–*	*22*.*1*	7.4	*20*.*2*	1.7	*1*.*1*	7.7	*20*.*7*
Internal primary branch	12	7.1	–	11.1	*21*.*5*	*–*	*25*.*9*	8.4	*23*.*0*	1.2	*1*.*4*	8.5	*22*.*8*
Internal secondary branch	9	5.2	–	9.0	*18*.*2*	*–*	*20*.*9*	6.8	*19*.*3*	1.3	*1*.*0*	7.3	*19*.*6*
Claw 2 lengths							* *		* *		* *		
External primary branch	10	7.4	–	12.6	*22*.*4*	*–*	*27*.*1*	9.5	*26*.*1*	1.5	*1*.*4*	9.9	*26*.*6*
External secondary branch	7	5.2	–	8.3	*17*.*3*	*–*	*22*.*3*	7.1	*20*.*3*	1.3	*1*.*8*	7.9	*21*.*2*
Internal primary branch	10	6.9	–	12.0	*20*.*0*	*–*	*26*.*4*	8.7	*24*.*1*	1.5	*1*.*8*	9.4	*25*.*3*
Internal secondary branch	8	5.2	–	10.0	*17*.*7*	*–*	*21*.*0*	7.0	*19*.*7*	1.5	*1*.*2*	7.8	*21*.*0*
Claw 3 lengths							* *		* *		* *		
External primary branch	11	7.3	–	13.1	*23*.*4*	*–*	*27*.*3*	9.5	*26*.*4*	1.7	*1*.*1*	10.0	*26*.*9*
External secondary branch	9	5.4	–	10.5	*16*.*6*	*–*	*23*.*6*	7.7	*21*.*0*	1.7	*2*.*1*	8.3	*22*.*3*
Internal primary branch	12	6.7	–	12.1	*20*.*2*	*–*	*26*.*0*	8.9	*24*.*4*	1.5	*1*.*6*	9.6	*25*.*8*
Internal secondary branch	9	5.1	–	8.7	*17*.*7*	*–*	*21*.*2*	7.0	*19*.*5*	1.4	*1*.*2*	7.9	*21*.*2*
Claw 4 lengths							* *		* *		* *		
Anterior primary branch	10	7.4	–	14.1	*24*.*6*	*–*	*29*.*4*	10.0	*27*.*5*	2.0	*1*.*5*	10.4	*28*.*0*
Anterior secondary branch	10	6.5	–	10.1	*18*.*5*	*–*	*24*.*2*	7.8	*21*.*6*	1.1	*1*.*7*	8.1	*21*.*8*
Posterior primary branch	12	8.0	–	13.7	*25*.*6*	*–*	*29*.*7*	10.5	*28*.*2*	1.8	*1*.*4*	10.8	*29*.*0*
Posterior secondary branch	11	5.0	–	9.6	*17*.*8*	*–*	*21*.*6*	7.6	*19*.*8*	1.4	*1*.*4*	8.0	*21*.*5*

**Table 7:** Measurements [in μm] of selected morphological structures of eggs of *Mesobiotus occultatus* sp. nov. mounted in Hoyer’s medium (N—number of specimens/structures measured, RANGE refers to the smallest and the largest structure among all measured eggs; SD—standard deviation).

CHARACTER	N	RANGE			MEAN	SD
Egg bare diameter	19	65.9	–	90.5	79.9	7.6
Egg full diameter	19	97.4	–	126.6	114.1	9.3
Process height	81	14.1	–	21.8	17.9	1.8
Process base width	81	13.1	–	20.0	16.0	1.7
Process base/height ratio	81	74%	–	106%	90%	7%
Distance between processes	78	1.4	–	4.2	2.6	0.6
Number of processes on the egg circumference	26	13	–	16	14.4	1.0

#### Material examined

**Type material:** Holotype (animal) and 50 paratypes (24 animals and 26 eggs).

**Additional material: I) Svalbard, Spitsbergen, Hornsund, Revdalen**: **1)** 77°01'41''N; 15°22'21''E, 67 m asl, moss on soil, northern part of the Revdalen, near the Revvatnet and the Revelva (1 egg); **2)** 77°01'39''N; 15°22'47''E, 76 m asl, moss on rock, northern part of the Revdalen, near the Revvatnet and the Revelva (5 animals and 2e); **3)** 77°01'09''N; 15°24'34''E, 50 m asl, moss on soil, northern part of the Revdalen, near the Revvatnet (southern edge) and the Revelva (1a and 4e); **4)** (77°01'09''N; 15°24'34''E), 50 m asl, moss and lichen on soil, northern part of the Revdalen, near the Revvatnet (southern edge) and the Revelva (4a and 5e) [65]; **II) Svalbard, Spitsbergen, Hornsund, Rotjesfjellet: 1)** 77°00'16''N; 15°24'02''E, 50 m asl, moss on soil, south-east slope (2e); **2)** 77°00'19''N; 15°23'55''E, 100 m asl, moss on soil, south-east slope (1a and 5e); **3)** 77°00'31''N; 15°23'21''E, 301 m asl, moss on soil, south-east slope (2a and 1e) [65]; **III) Svalbard, Spitsbergen, Hornsund, Ariekammen: 1)** 77°01'10''N; 15°31'16''E, 524 m asl, moss on rock (20a and 3e); **2)** 77°00'31''N; 15°31'43''E, 50 m asl, moss on rock (7a and 12e); **2)** 77°00'18''N; 15°32'01''E, 14 m asl, moss on rock (6a and 1e) [66]; **3)** 77°00'48''N; 15°33'05''E, 11 m asl, moss on rock (48a and 70e); **4)** 77°00'29''N; 15°33'09''E, 1 m asl, moss on rock (22a and 3e); **5)** 77°00'40''N; 15°32'88''E, 7 m asl, moss on soil (1e) **6)** 77°00'58''N; 15°32'00''E, 28 m asl, moss on soil (1a and 1e); **7)** 77°00'50''N; 15°33'24''E, 8 m asl, lichen on stone (6a and 2e) **8)** 77°00'50''N; 15°33'24''E, 8 m asl, moss and lichen on stone (6a and 5e), **9)** 77°00'79''N; 15°32'66''E, 72 m asl, moss and lichen on stone (2a and 1e), **10)** 77°00'79''N 15°32'66''E, 72 m asl, moss on stone (1a and 1e) [67]; **IV) Svalbard, Phippsøya:** 80°41.211’N; 20°50.606’E, 47 m asl, moss on rock (6a and 15e) [72].

#### Description of *Mesobiotus occultatus* sp. nov

**Animals (morphometrics in Table 6).** Body white in living specimens and transparent after fixation (Fig 8A). Eyes present. Cuticle smooth, *i*.*e*., without gibbosities, papillae, spines, sculpture or pores. However, under SEM microgranulation is visible on the entire dorso-lateral cuticle (Fig 8B). Granulation present on the external surface of all legs (Fig 8C–8E).

Bucco-pharyngeal apparatus of the *Macrobiotus* type (Fig 9A), with the ventral lamina and ten peribuccal lamellae. Mouth antero-ventral. The oral cavity armature well developed and composed of three bands of teeth (Fig 9B and 9C). The first band of teeth is composed of numerous small granules arranged in a several rows situated anteriorly in the oral cavity, just behind the bases of the peribuccal lamellae (Fig 9B and 9C, arrowhead). The band is hardly detectible under PCM in small specimens but clearly visible in large individuals. The second band of teeth is situated between the ring fold and the third band of teeth and comprises of ridges parallel to the main axis of the buccal tube (Fig 9B, arrow). The teeth of the third band are located within the posterior portion of the oral cavity, between the second band of teeth and the buccal tube opening (Fig 9B and 9C, indented arrowhead). The third band of teeth is divided into the dorsal and the ventral portion. Under PCM, both dorsal and ventral teeth are visible as two lateral and one median transverse ridges (Fig 9B, indented arrowhead). Pharyngeal bulb spherical, with triangular apophyses, three rod-shaped macroplacoids and a triangular microplacoid. Macroplacoid length sequence 2<3≤1. The first macroplacoid narrower anteriorly, the second without constrictions and the third with a small, subterminal constriction (Fig 9D, empty arrowhead).

Claws of the *Mesobiotus* type (Fig 10A–10C). Primary branches with distinct accessory points. Lunules under all claws smooth (Fig 10A–10C). Thin cuticular bars under claws I–III present (Fig 10A, arrow). Other cuticular structures on legs absent.

**Eggs (morphometrics in Table 7).** Laid freely, white, spherical and ornamented (Fig 11A and 11B). Egg processes in the shape of wide cones. The cones are sometimes bifurcated on the top and often with one to few short apical filaments (Fig 12A–12H). In PCM, processes reticulated with mesh size 0.5–1.6 μm in diameter, slightly larger near the process base (Fig 12A–12D). In some processes larger meshes are present in the apical part of the process (up to 3.5 μm in diameter; Fig 12A, arrow). At the base of each process a crown of small but well visible thickenings visible both in PCM and SEM (Fig 11C, arrows). In SEM, processes smooth, but with well visible small pores at the processes bases (Fig 12E and 12F and 12H, arrows). Very fine granulation present at the apical part of each process (Fig 12E–12H, empty arrowhead). Egg areolation absent (Fig 11A–11D). In PCM, the surface between processes with irregular and rather poorly visible dots (Fig 11C), in SEM visible as large ridges (Fig 11D).

*DNA sequences*: We obtained sequences for three of the above mentioned molecular markers from five of six analysed paragenophores (see Table 1 for details). All markers were represented by single haplotypes:

The **18S rRNA** sequence (GenBank: MH197147), 811 bp long:

The **ITS-2 rRNA** sequence (GenBank: MH197155), 419 bp long:

The **COI** sequence (GenBank: MH195152), 575 bp long:

**Etymology.** The name “*occultatus*”, from Latin = hidden, is given to the new species, because the species remained unrecognised until the nominal *M*. *harmsworthi* was accurately characterised.

**Type Locality.** Norway; 77°00'48''N; 15°33'05''E, 11 m asl, Svalbard, Spitsbergen, Hornsund, vicinity of Polish Polar Station “Hornsund”, Ariekammen, southern slope, moss on rock.

**Type depositories.** Holotype (slide SV83.7/5) and 25 paratypes (14 specimens and 11 eggs) (slides SV83.7/2, SV83.7/5, SV83.7/8, SV83.7/9, SV83.7/10, SV83.7/12, SV83.7/13, SV83.7/14, SV83.7/15, SV83.7/16) are deposited at the Department of Animal Taxonomy and Ecology, Institute of Environmental Biology, Adam Mickiewicz University, Poznań, Umultowska 89, 61–614 Poznań (Poland); 12 paratypes (6 specimens and 6 eggs) (slides SV83.7/4, KZ83.7/11) are deposited at the Institute of Zoology and Biomedical Research, Jagiellonian University, Gronostajowa 9, 30–387, Kraków, Poland; 10 paratypes (3 animals and 7 eggs) (slides SV83.7/1, SV83.7/6) are deposited at the Zoological Museum, Natural History Museum of Denmark, University of Copenhagen, Universitetsparken 15, DK-2100 Copenhagen Ø, Denmark; 3 paratypes (1 animals and 2 eggs) (slide SV83.7/3) are deposited at the collection of Binda and Pilato in Department of Animal Biology “Marcello La Greca”, University of Catania, Italy.

**Phenotypic differential diagnosis.** The new species by the shape of egg processes and surface of eggs is the most similar to *M*. *baltatus* (McInnes, 1991) [31], *M*. *coronatus* (de Barros, 1942) [14], *M*. *insuetus* (Pilato, Sabella & Lisi, 2014) [33], *M*. *patiens* (Pilato, Binda, Napolitano, & Moncada, 2000) [13], *M*. *pseudocoronatus* (Pilato, Binda & Lisi, 2006) [73], *M*. *pseudopatiens* and *M*. *simulans* (Pilato, Binda, Napolitano, & Moncada, 2000) [13] but differs specifically from:

1. ***M*. *baltatus*** (known only from type locality in Spain [31]) by: cuticle without brown bands of pigmentation, the presence of occasional short filaments on apices of egg processes, the presence of large meshes near the apices of egg processes and by a larger number of processes on the egg circumference (13–16 in the new species *vs ca*. 12 in *M*. *baltatus*).
2. ***M*. *coronatus*** (known from few localities in South America [69]) by: the absence of supplementary teeth in the oral cavity, a different macroplacoid length sequence (2<3≤1 in the new species *vs* 2<3<1 in *M*. *coronatus*), much less evident crown of thickenings around egg processes, the presence of short filaments on top of some egg processes, the presence of large meshes near the top of egg processes, a larger diameter of eggs without and with processes (65.9–90.5 μm and 97.4–126.6 μm respectively, in the new species *vs* 42.0–55.0 μm and 55.0–71.0 μm respectively, in *M*. *coronatus*), larger egg processes (14.1–21.8 μm in the new species *vs ca*. 9.2 μm in *M*. *coronatus*) and by wider process bases (13.1–20.0 μm in the new species *vs* 9.6–10.4 μm in *M*. *coronatus*).
3. ***M*. *insuetus*** (known only from type locality in Italy [33]) by: the presence of eyes, typically developed claws IV (the basal tract of posterior and anterior claws much longer, primary and secondary branches forming an almost 90° angle in *M*. *insuetus*), the occasional presence of short filaments on egg process apices, the presence of large meshes the apical part of egg processes, a larger diameter of eggs with processes (97.4–126.6 μm in the new species *vs* 73.0–86.2 μm in *M*. *insuetus*) and by higher egg processes (14.1–21.8 μm in the new species *vs ca*. 7.9–8.6 μm in *M*. *insuetus*).
4. ***M*. *patiens*** (known from few localities in Italy [13]) by: the presence of eyes, a different macroplacoid length sequence (2<3≤1 in the new species *vs* 2<3<1 in *M*. *patiens*) and by the occasional presence of short filaments on egg process apices, and by the presence of large meshes in the apical part of egg processes.
5. ***M*. *pseudocoronatus*** (known only from type locality on Seychelles [73]) by: the presence of smooth dorsal cuticle (small tubercles present in *M*. *pseudocoronatus*), much less evident crown of thickenings around egg processes, the absence of dentated lunules under claws IV, the presence of large meshes in the apical area of egg processes and a by higher egg processes (14.1–21.8 μm in the new species *vs ca*. 10.9–12.7 μm in *M*. *pseudocoronatus*).
6. ***M*. *pseudopatiens*** (known only from type locality in Costa Rica [38] by: the presence of eyes, the presence of granulation on legs I-III, the presence of the first band of teeth in oral cavity (visible under PCM in the new species *vs* invisible in *M*. *pseudopatiens*), a slightly different macroplacoid length sequence (2<3≤1in the new species *vs* 2<3<1 in *M*. *pseudopatiens*), the presence of large meshes in the apical area of egg processes, the absence of a long, flexible terminal part of egg process, a larger bare and full egg diameter (65.9–90.5 μm and 97.4–126.6 μm respectively, in the new species *vs* 55.5–59.3 μm and 80.4–88.0 μm respectively, in *M*. *pseudopatiens*) and by a larger number of processes on the egg circumference (13–16 in the new species *vs* 11–12 in *M*. *pseudopatiens*).
7. ***M*. *simulans*** (known from few localities in Italy [13]) by: the absence of dentated lunules under claws IV, a much less evident crown of thickenings around egg processes, the occasional presence of short filaments on egg process apices, the presence of large meshes in the apical area of egg process, and by a larger egg processes (14.1–21.8 μm in the new species *vs ca*. 11.0 μm in *M*. *simulans*).
8. **Genotypic differential diagnosis.** The ranges of uncorrected genetic p-distances between the new species and species of the genus *Mesobiotus*, for which sequences are available from GenBank, were as follows:
9. **18S rRNA**: 0.4–5.5% (3.4% on average), with the most similar being *M*. *harmsworthi* from Spitsbergen (MH197146) and the least similar being *M*. cf. *mottai* and *M*. *furciger* from the Antarctic (KT226072 and EU266928 respectively);
10. **ITS-2**: 9.0–30.0% (19.4% on average), with the most similar being undetermined *M*. *harmsworthi* species from Russia (MH197157) and the least similar being an undetermined *M*. *furciger* group species from Norway (MH197156);
11. **COI**: 18.2–24.8% (20.7% on average), with the most similar being an undetermined *M*. *furciger* group species from Norway (MH195153) and the least similar *M*. *furciger* from the Antarctic (JX865308).

### *Mesobiotus skorackii* sp. nov

**Fig 13:**
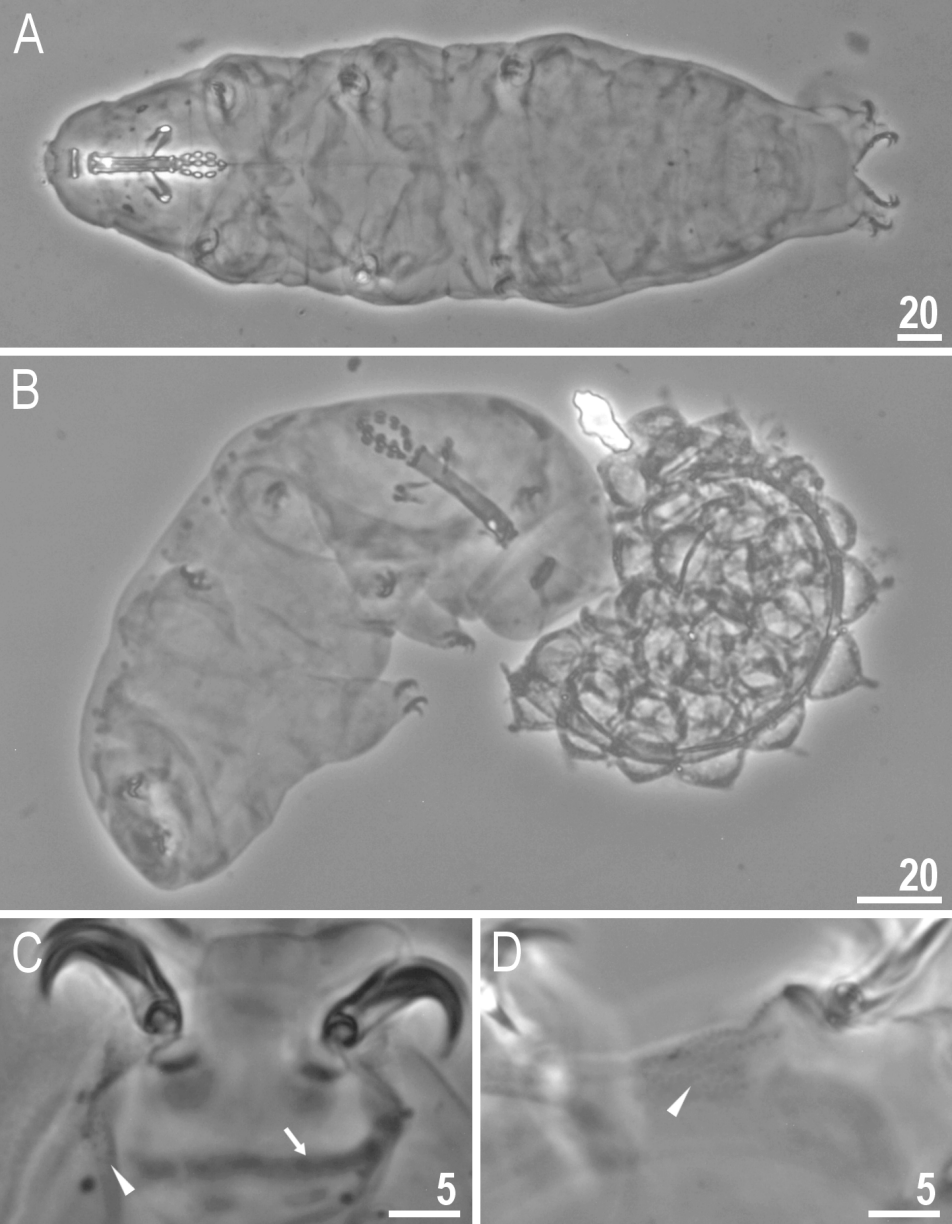
*Mesobiotus skorackii* sp. nov.–habitus and granulation on legs. A–dorso-ventral projection of the entire animal (holotype); B–a young specimen hatches from the egg (paratype); C–poorly visible granulation on leg II, arrowhead (paratype); arrow indicates cuticular bar under claws; D–granulation on leg IV, arrowhead (paratype). Scale bars in micrometres [μm]. All PCM.

**Fig 14:**
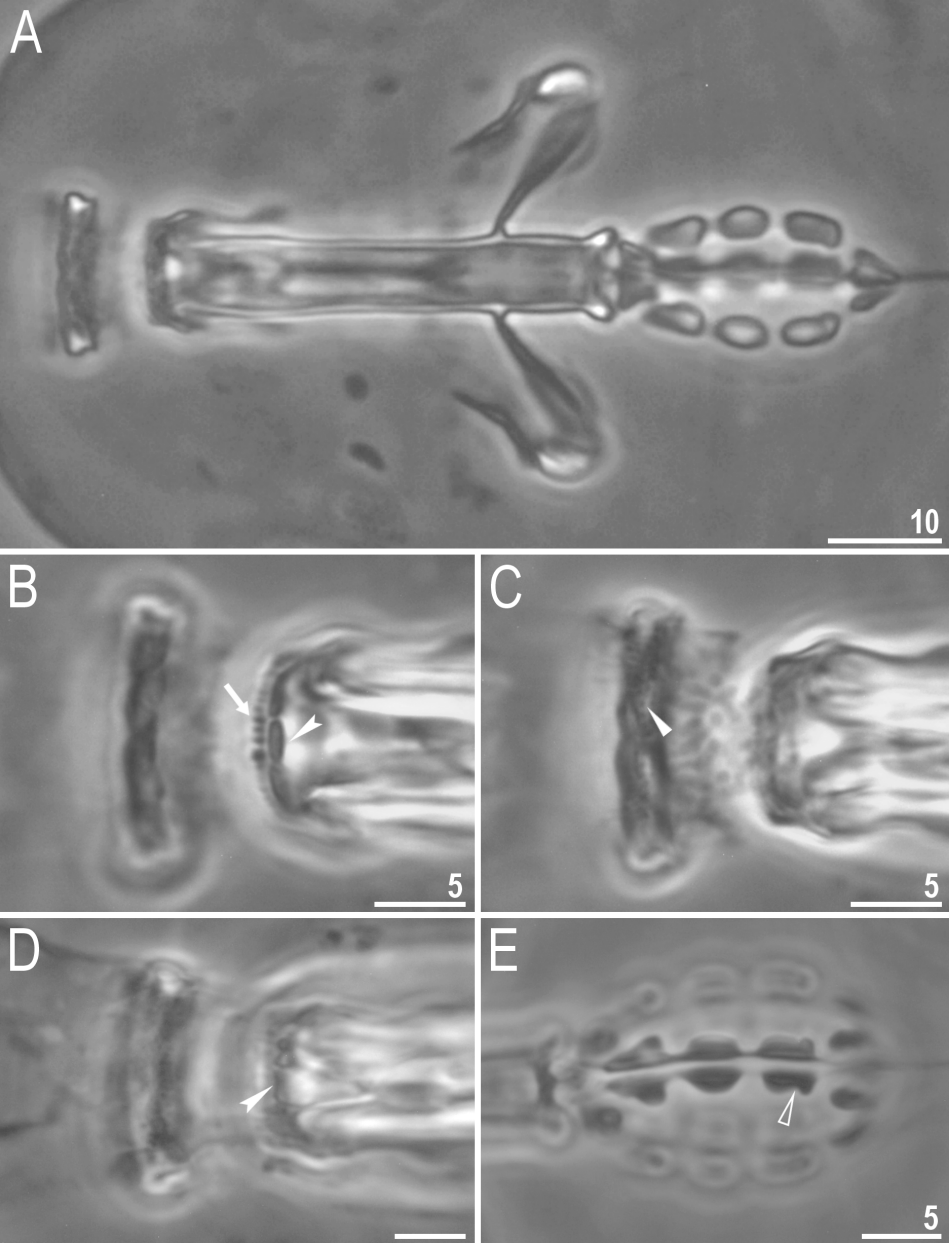
*Mesobiotus skorackii* sp. nov.—buccal apparatus and the oral cavity armature. A–general view (holotype); B–D–oral cavity armature; the filled flat arrowhead indicates teeth of the first band, the arrow indicates teeth of the second band, indented arrowheads indicates teeth of the third band (holotype); E–ventral placoids; the empty arrowhead indicates a subterminal constriction (holotype). Scale bars in micrometres [μm]. All PCM.

**Fig 15:**
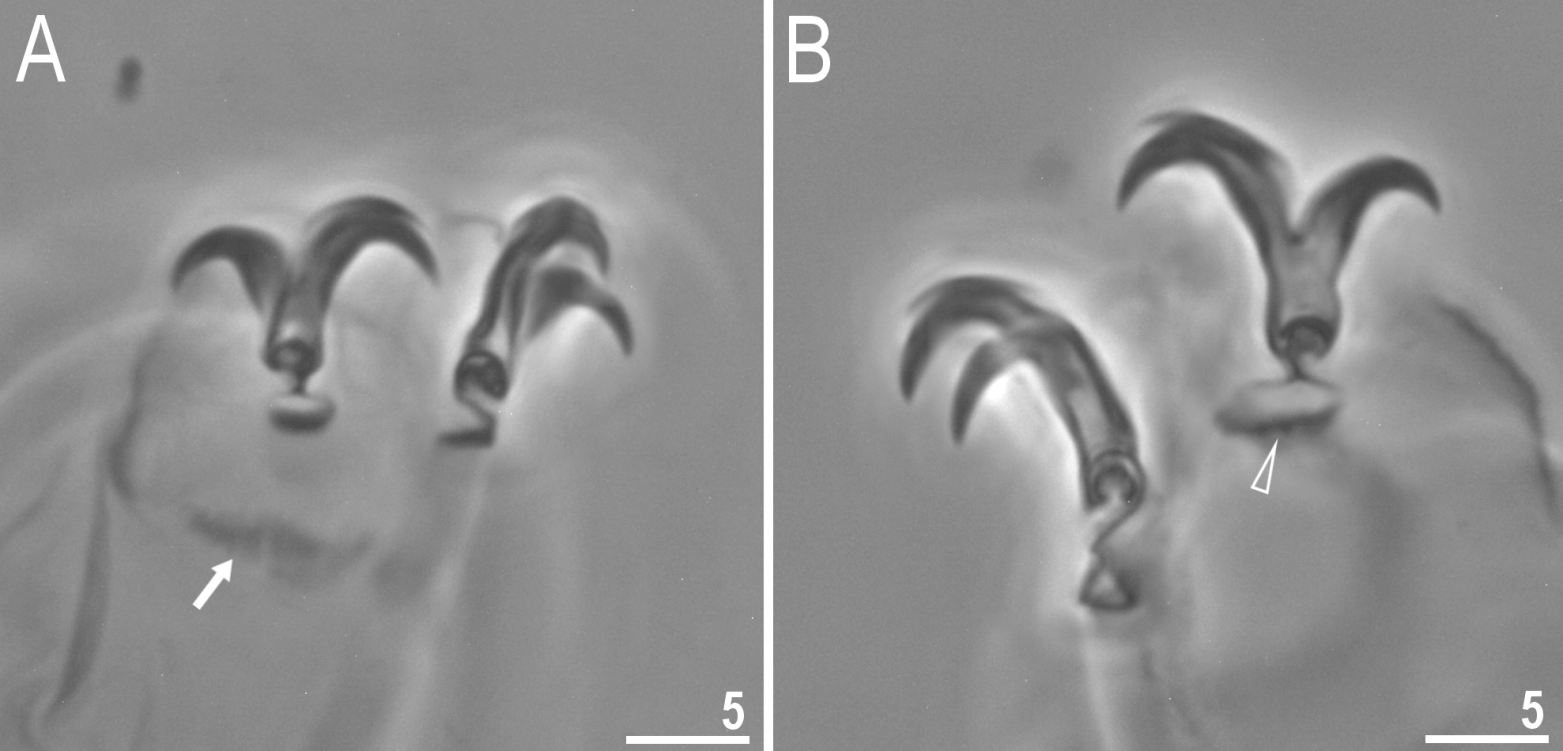
*Mesobiotus skorackii* sp. nov.–claws. A–claws III with smooth lunules; arrow indicates cuticular bar under claws (paratype); B–claws IV with indented lunules (empty arrowhead) (holotype). Scale bars in micrometres [μm]. All PCM.

**Fig 16:**
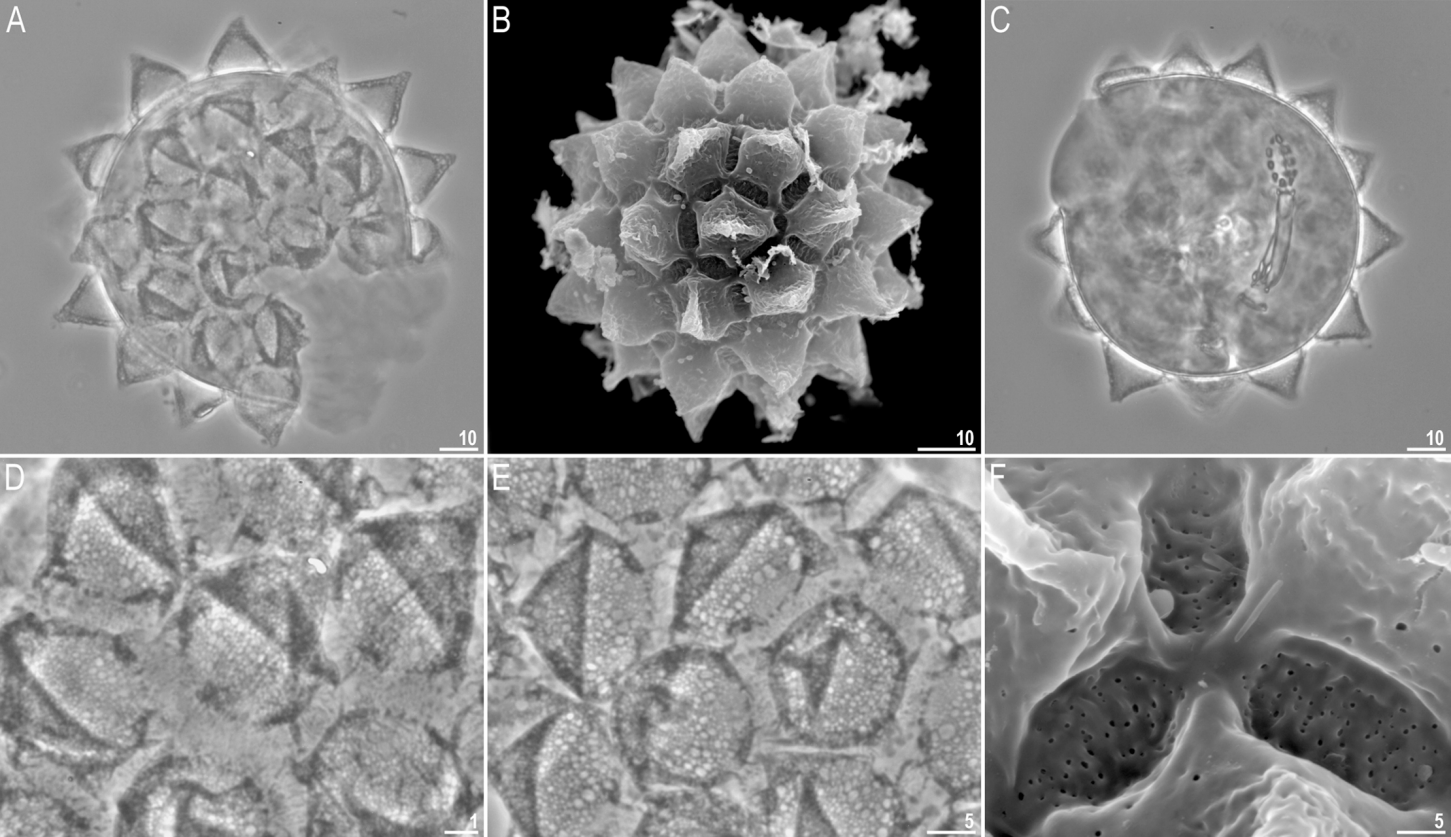
*Mesobiotus skorackii* sp. nov.–eggs. A–C–egg chorion visible in PCM and SEM; D–E–the surface between egg processes visible in PCM; F–the surface between egg processes visible in SEM. Scale bars in micrometres [μm].

**Fig 17:**
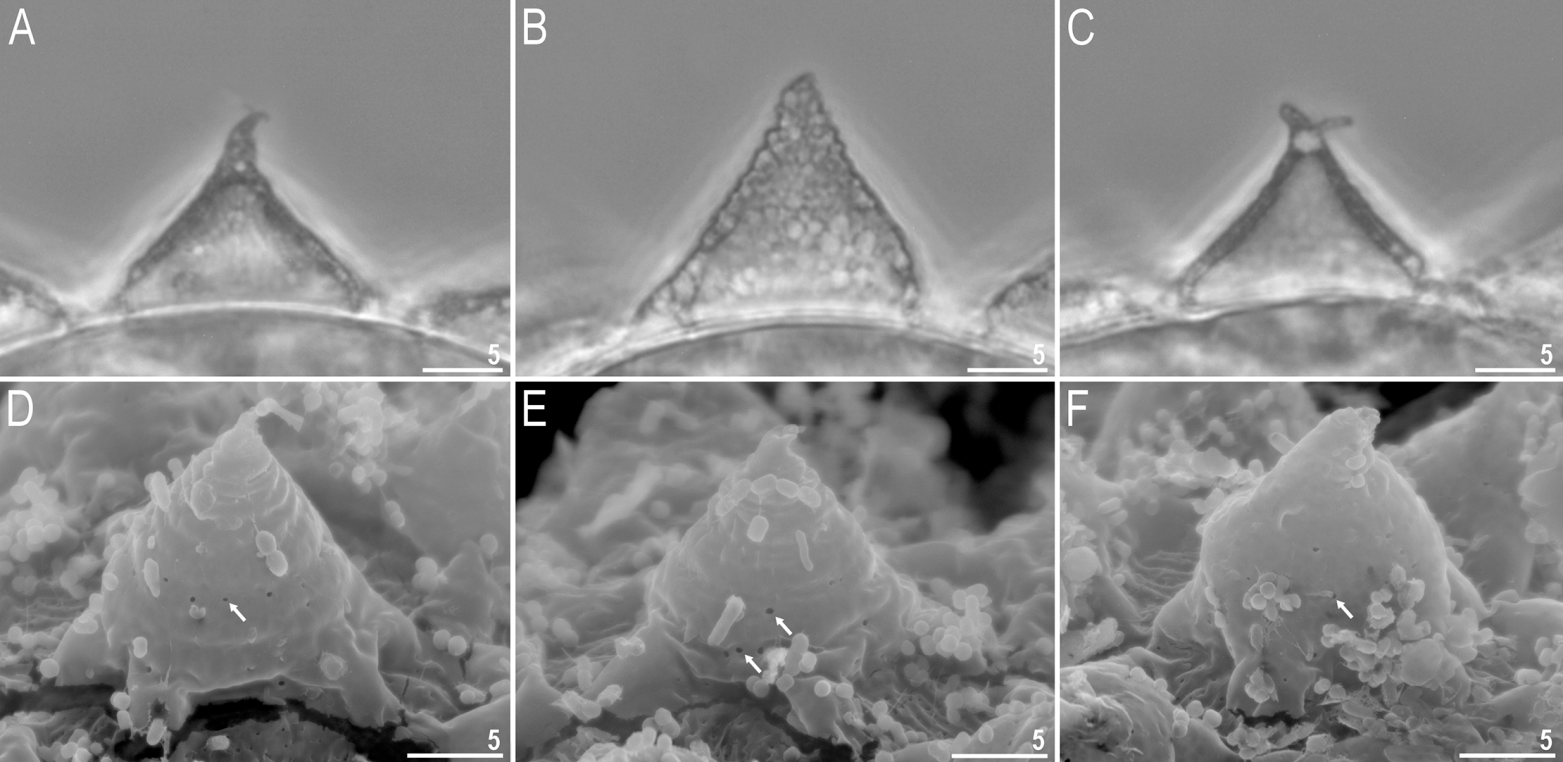
*Mesobiotus skorackii* sp. nov.–egg processes morphology. A–C–egg processes morphology seen in PCM; D–F–egg processes morphology seen in SEM; arrows indicate small pores at the processes base. Scale bars in micrometres [μm].

urn:lsid:zoobank.org:act:3C14C322-09B8-470C-B5BB-7C30C2EE4A5B

(Figs 13–17; Tables 8 and 9)

**Table 8:** Measurements and *pt* values of selected morphological structures of *Mesobiotus skorackii* sp. nov. mounted in Hoyer’s medium (N—number of specimens/structures measured, RANGE refers to the smallest and the largest structure among all measured specimens; SD—standard deviation).

CHARACTER	N	RANGE						MEAN		SD		Holotype	
		μm			*pt*			μm	*pt*	μm	*pt*	μm	*pt*
Body length	25	206	–	440		*–*		351	*–*	66	*–*	378	*–*
Buccal tube													
Buccal tube length	25	26.6	–	47.1		–		38.3	*–*	4.7	*–*	40.0	*–*
Stylet support insertion point	25	20.4	–	36.0	*76*.*0*	*–*	*79*.*6*	29.8	*77*.*6*	3.7	*1*.*0*	30.9	*77*.*3*
Buccal tube external width	25	4.3	–	7.8	*15*.*6*	*–*	*17*.*8*	6.4	*16*.*7*	0.9	*0*.*6*	7.0	*17*.*5*
Buccal tube internal width	25	2.9	–	6.0	*10*.*5*	*–*	*13*.*0*	4.5	*11*.*7*	0.7	*0*.*7*	5.0	*12*.*5*
Ventral lamina length	25	17.0	–	30.0	*61*.*1*	*–*	*64*.*9*	24.2	*63*.*0*	3.0	*1*.*2*	25.0	*62*.*5*
Placoid lengths													
Macroplacoid 1	25	3.5	–	6.5	*12*.*1*	*–*	*15*.*1*	5.3	*13*.*7*	0.8	*0*.*8*	5.3	*13*.*3*
Macroplacoid 2	25	2.7	–	5.1	*9*.*7*	*–*	*11*.*8*	4.2	*10*.*9*	0.7	*0*.*6*	4.4	*11*.*0*
Macroplacoid 3	25	2.9	–	6.1	*10*.*9*	*–*	*13*.*9*	4.9	*12*.*6*	0.8	*0*.*8*	5.1	*12*.*8*
Microplacoid	25	2.5	–	4.8	*9*.*1*	*–*	*11*.*1*	3.9	*10*.*1*	0.6	*0*.*6*	4.2	*10*.*5*
Macroplacoid row	25	10.8	–	21.2	*40*.*6*	*–*	*46*.*4*	16.8	*43*.*6*	2.5	*1*.*7*	17.7	*44*.*3*
Placoid row	25	13.2	–	27.6	*49*.*6*	*–*	*59*.*1*	21.5	*55*.*8*	3.3	*2*.*4*	22.4	*56*.*0*
Claw 1 lengths													
External primary branch	23	6.3	–	11.7	*22*.*3*	*–*	*27*.*0*	9.4	*24*.*5*	1.4	*1*.*1*	9.9	*24*.*8*
External secondary branch	15	5.7	–	9.0	*17*.*6*	*–*	*23*.*3*	7.6	*19*.*6*	1.0	*1*.*6*	7.3	*18*.*3*
Internal primary branch	23	6.1	–	10.9	*19*.*4*	*–*	*24*.*7*	8.8	*22*.*8*	1.3	*1*.*2*	9.2	*23*.*0*
Internal secondary branch	18	5.7	–	8.7	*16*.*4*	*–*	*21*.*6*	7.4	*18*.*7*	0.9	*1*.*5*	7.0	*17*.*5*
Claw 2 lengths													
External primary branch	21	7.0	–	12.9	*21*.*3*	*–*	*27*.*4*	9.6	*25*.*1*	1.4	*1*.*5*	10.4	*26*.*0*
External secondary branch	14	6.1	–	9.1	*17*.*6*	*–*	*23*.*6*	7.8	*20*.*4*	1.1	*1*.*8*	8.6	*21*.*5*
Internal primary branch	22	6.5	–	10.9	*19*.*9*	*–*	*24*.*4*	8.7	*22*.*7*	1.2	*1*.*2*	9.0	*22*.*5*
Internal secondary branch	11	4.9	–	9.4	*16*.*6*	*–*	*21*.*7*	7.0	*18*.*8*	1.4	*1*.*5*	7.2	*18*.*0*
Claw 3 lengths							* *		* *		* *		
External primary branch	22	7.1	–	12.0	*21*.*0*	*–*	*28*.*2*	9.8	*25*.*6*	1.3	*1*.*4*	10.5	*26*.*3*
External secondary branch	18	6.2	–	8.9	*18*.*1*	*–*	*23*.*3*	7.5	*20*.*3*	1.0	*1*.*5*	8.4	*21*.*0*
Internal primary branch	22	6.9	–	10.4	*20*.*2*	*–*	*26*.*3*	8.9	*23*.*3*	1.1	*1*.*3*	9.6	*24*.*0*
Internal secondary branch	15	5.5	–	9.4	*16*.*9*	*–*	*21*.*7*	7.4	*19*.*3*	1.1	*1*.*4*	7.0	*17*.*5*
Claw 4 lengths													
Anterior primary branch	20	7.7	–	14.8	*24*.*3*	*–*	*31*.*4*	11.0	*28*.*1*	1.8	*2*.*1*	11.9	*29*.*8*
Anterior secondary branch	19	6.1	–	11.7	*19*.*0*	*–*	*24*.*8*	8.8	*22*.*6*	1.5	*1*.*7*	9.2	*23*.*0*
Posterior primary branch	20	8.7	–	15.0	*25*.*3*	*–*	*32*.*5*	11.4	*29*.*8*	1.8	*1*.*9*	12.8	*32*.*0*
Posterior secondary branch	19	6.1	–	11.0	*17*.*8*	*–*	*23*.*7*	8.2	*21*.*4*	1.5	*1*.*9*	9.1	*22*.*8*

**Table 9:** Measurements [in μm] of selected morphological structures of eggs of *Mesobiotus skorackii* sp. nov. mounted in Hoyer’s medium (N—number of specimens/structures measured, RANGE refers to the smallest and the largest structure among all measured eggs; SD—standard deviation).

CHARACTER	N	RANGE			MEAN	SD
Egg bare diameter	16	66.8	–	81.0	76.3	3.8
Egg full diameter	16	89.2	–	103.8	97.8	4.3
Process height	69	9.5	–	16.0	12.2	1.5
Process base width	69	13.6	–	21.3	17.3	1.7
Process base/height ratio	69	123%	–	175%	142%	12%
Distance between processes	69	2.0	–	4.4	3.0	0.5
Number of processes on the egg circumference	23	10	–	12	11.7	0.6

#### Material examined

**Type material:** Holotype (animal) and 64 paratypes (37 animals and 27 eggs).

**Additional material: I) Chuy Region, Alamudun District, Ala Archa National Park:** 46) 42°39′N, 74°28′E, *ca*. 2000 m asl, spruce forest, lichen on rock (15 animals and 3 eggs); **II) Issyk-Kul Region, Issyk-Kul District, Issyk Kul Biosphere Reserve, near Grigorievka:** 8) 42°47′N, 77°28′E, *ca*. 2000 m asl, spruce forest, moss on rock (3a and 1e); **III) Issyk-Kul Region, Issyk-Kul District, Ak-Suu Valley, northern slope:** 26) 42°51′N, 77°18′E, 3100 m asl, spruce forest, moss from tree (22a and 7e); 72) 42°53′N, 77°16′E, 3900 m asl, above forest line, moss on rock (21a and 24e); 81) 42°52′N, 77°16′E, 3650 m asl, above forest line, moss on rock (2a and 1e); 82) 42°52′N, 77°16′E, 4000 m asl, above forest line, moss on rock (1a and 2e); 83) 42°52′N, 77°16′E, 4000 m asl, above forest line, moss on rock (4a and 1e); 85) 42°52′N, 77°16′E, 3600 m asl, above forest line, moss on rock (15a and 13e); 89) 42°52′N, 77°16′E, 3450 m asl, above forest line, moss on rock (2a and 9e); 91) 42°52′N, 77°16′E, 3600 m asl, above forest line, moss on rock (10a and 2e); 93) 42°52′N, 77°16′E, 4000 m asl, above forest line, moss on rock (5a and 1e); 100) 42°52′N, 77°16′E, 3700 m asl, above forest line, moss on rock (2a and 1e); 102) 42°52′N, 77°16′E, 4000 m asl, above forest line, moss on rock (3s and 2e).

#### Description of *Mesobiotus skorackii* sp. nov

**Animals (morphometrics in Table 8).** Body white in living specimens and transparent after fixation (Fig 13A and 13B). Eyes present in 48% studied specimens (see also comments in Remarks below). Cuticle smooth, *i*.*e*., without gibbosities, papillae, spines, sculpture or pores. Granulation present on the external surface of all legs. On legs I–III granulation is hardly visible only in some specimens, whereas granulation on legs IV always clearly visible (Fig 13C and 13D, arrowheads).

Bucco-pharyngeal apparatus of the *Macrobiotus* type (Fig 14A), with the ventral lamina and ten peribuccal lamellae. Mouth antero-ventral. The oral cavity armature well developed and composed of three bands of teeth (Fig 14B–14D). The first band of teeth is composed of numerous small granules arranged in a several rows situated anteriorly in the oral cavity, just behind the bases of the peribuccal lamellae (Fig 14C, arrowhead). The band is hardly detectible under PCM in small specimens and clearly visible in large individuals. The second band of teeth is situated between the ring fold and the third band of teeth and comprises of ridges parallel to the main axis of the buccal tube larger than those in the first band (Fig 14B, arrow). The teeth of the third band are located within the posterior portion of the oral cavity, between the second band of teeth and the buccal tube opening (Fig 14B and 14D, indented arrowhead). The third band of teeth is divided into the dorsal and the ventral portion. Under PCM, both dorsal and ventral teeth are visible as two lateral and one median transverse ridges (Fig 14B and 14D, indented arrowhead). Pharyngeal bulb spherical, with triangular apophyses, three rod-shaped macroplacoids and a triangular microplacoid. Macroplacoid length sequence 2<3<1. The first macroplacoid narrower anteriorly, the second without constrictions and the third with a small, subterminal constriction (Fig 14E, empty arrowhead).

Claws of the *Mesobiotus* type (Fig 15A and 15B). Primary branches with distinct accessory points. Lunules under claws I–III smooth and slightly dentated under claws IV (Fig 15B, empty arrowhead). Thin cuticular bars under claws I–III present (Figs 13C and 15A, arrow). Other cuticular structures on legs absent.

**Eggs (morphometrics in Table 9).** Laid freely, white, spherical and ornamented, with processes and delicate areolation (Fig 16A–16C). Egg processes in the shape of short and wide sharpened cones (Figs 16A, 16B and 17A–17F). In PCM, processes reticulated with mesh size 0.3–1.8 μm in diameter, slightly increased in size from the base to the top (Fig 17B). In SEM processes smooth, but with well visible small pores present mainly at the processes bases (Fig 17D–17F, arrows). Each process surrounded by six areolae delimited by thin brims (Fig 16B and 16D–16F). The brims are very often discontinuous, thus areolae are not always fully formed (Fig 16D and 16E). Surface inside the areolae with clearly visible wrinkles, in PCM (Fig 16D and 16E), and small pores and wrinkles in SEM (Fig 16F).

**Etymology.** We dedicate this species to our friend and a distinguished and prominent Polish acarologist, Professor Maciej Skoracki, a discoverer of many new species of Syringophilidae mites.

**Type Locality.** Kyrgyz Republic; 42°52′N, 77°16′E, 3500 m asl, Issyk-Kul Region, Issyk-Kul District, Ak-Suu Valley, northern slope, above forest line, moss on rock.

**Type depositories.** Holotype (slide KZ79/9) and 25 paratypes (13 animals and 12 eggs) (slides KZ79/6, KZ79/7, KZ79/9,) are deposited at the Department of Animal Taxonomy and Ecology, Institute of Environmental Biology, Adam Mickiewicz University, Poznań, Umultowska 89, 61–614 Poznań (Poland); 24 paratypes (17 animals and 7 eggs) (slides KZ79/1, KZ79/2) are deposited at the Institute of Zoology and Biomedical Research, Jagiellonian University, Gronostajowa 9, 30–387, Kraków, Poland; 7 paratypes (2 animals and 5 eggs) (slides KZ79/5, KZ79/8) are deposited at the Zoological Museum, Natural History Museum of Denmark, University of Copenhagen, Universitetsparken 15, DK-2100 Copenhagen Ø, Denmark; 7 paratypes (5 animals and 3 eggs) (slides KZ79/3, KZ79/4) are deposited at the collection of Binda and Pilato in Department of Animal Biology “Marcello La Greca”, University of Catania, Italy.

**Remarks.** Eyes were present in in all specimens in freshly made microscope slides (prepared less than 6 months ago) but are absent in all specimens mounted over 8 years ago. This probably means that eyes disappear with time in specimens mounted in Hoyer’s medium.

**Differential diagnosis.** The new species by the presence of smooth cuticle and egg areolation (sometimes not fully developed) is most similar to *M*. *barbarae*, *M*. *ethiopicus*, *M*. *harmsworthi*, *M*. *hieronimi*, *M*. *nuragicus*, *M*. *ovostriatus*, *M*. *peterseni*, *M*. *pseudoliviae*, but differs specifically from:

1. ***M*. *barbarae*** by: the presence of an undivided ventro-median tooth of the third band of teeth, the absence of a flexible terminal portion of egg processes, the areoles not always fully formed on the egg surface, a different macroplacoid length sequence (2<3<1 in the new species *vs* 2<1<3 in *M*. *barbarae*), a smaller egg full diameter (89.2–103.8 μm in the new species *vs* 106.0–115.0 μm in *M*. *barbarae*) and by shorter egg processes (9.5–16.0 μm in the new species *vs* 18.4–26.5 μm in *M*. *barbarae*).
2. ***M*. *ethiopicus*** by: the presence of eyes, the absence of evidently larger teeth in the second band of teeth, a different morphology of egg process apices (absence of flexible filaments in the new species *vs* processes terminated by several short, thin, and flexible filaments susceptible to fracture in *M*. *ethiopicus*) and by a higher process base/height ratio (123–175% in the new species *vs* 77–117% in *M*. *ethiopicus*)
3. ***M*. *harmsworthi*** (with the only confirmed localities being in the Svalbard Archipelago; for more details see Discussion below) by: the absence of additional teeth in second band of teeth, the absence of large and protruding accessory points on claws IV and by reticulation of egg process (meshes slightly increasing in size from the base to the top of the process in new species *vs* evidently larger meshes near the bases and apices of egg processes in *M*. *harmsworthi*).
4. ***M*. *hieronimi*** by: the teeth in second band of teeth never joined, the presence of dentated lunules under claws IV, a more posterior position of stylet supports (*pt = 76*.*0–79*.*6* in the new species *vs pt = 73*.*3–74*.*8* in *M*. *hieronimi*), the areoles not always fully formed on the egg surface, a smaller egg full diameter (89.2–103.8 μm in the new species *vs* 104.0–120.0 μm in *M*. *hieronimi*) and by smaller egg processes (9.5–16.0 μm in the new species *vs* 25.0–34.0 μm in *M*. *hieronimi*).
5. ***M*. *nuragicus*** by: a different macroplacoid length sequence (2<3<1 in the new species *vs* 2<1<3 in *M*. *nuragicus*), undivided egg processes apices (branched into a few short filaments in *M*. *nuragicus*), the areoles not always fully formed on the egg surface, a smaller egg full diameter (89.2–103.8 μm in the new species *vs ca*. 106.0 μm in *M*. *nuragicus*) and by shorter egg processes (9.5–16.0 μm in the new species *vs ca*. 17.0 μm in *M*. *nuragicus*).
6. ***M*. *ovostriatus*** by: the presence of the first band of teeth (in PCM), a better developed second band of teeth (second band of teeth reduced and composed of small granular teeth in *M*. *ovostriatus*), slightly dentated lunules under claws IV (smooth in *M*. *ovostriatus*), the absence of the long and flexible terminal portion of egg processes and by the areolae not always fully formed on the egg surface.
7. ***M*. *peterseni*** by: a different macroplacoid length sequence (2<3<1 in the new species vs 2<1<3 in *M*. *peterseni*) and by a different shape of egg processes (sharpened cones in the new species *vs* cones blunt and dome-shaped in *M*. *peterseni*).
8. ***M*. *pseudoliviae*** by: the teeth in second band of teeth never joined, the areoles not always fully formed on the egg surface, fewer areoles around the egg processes (6 in the new species *vs ca*. 16 in *M*. *pseudoliviae*), a smaller egg full diameter (89.2–103.8 μm in the new species *vs* 156.0–177.0 μm in *M*. *pseudoliviae*), shorter egg processes (9.5–16.0 μm in the new species *vs* 42.0–56.0 μm in *M*. *pseudoliviae*) and by narrower process bases (13.6–21.3 μm in the new species *vs* 28.0–45.0 μm in *M*. *pseudoliviae*). Important note: Maximum dimensions (both absolute and relative) of all morphological structures are smaller in *M*. *harmsworthi*, but the morphometric comparison of the two species must be treated with caution because only one specimen was measured in the original description of *M*. *pseudoliviae*.

#### Phylogeny and molecular species delimitation

**Fig 18:**
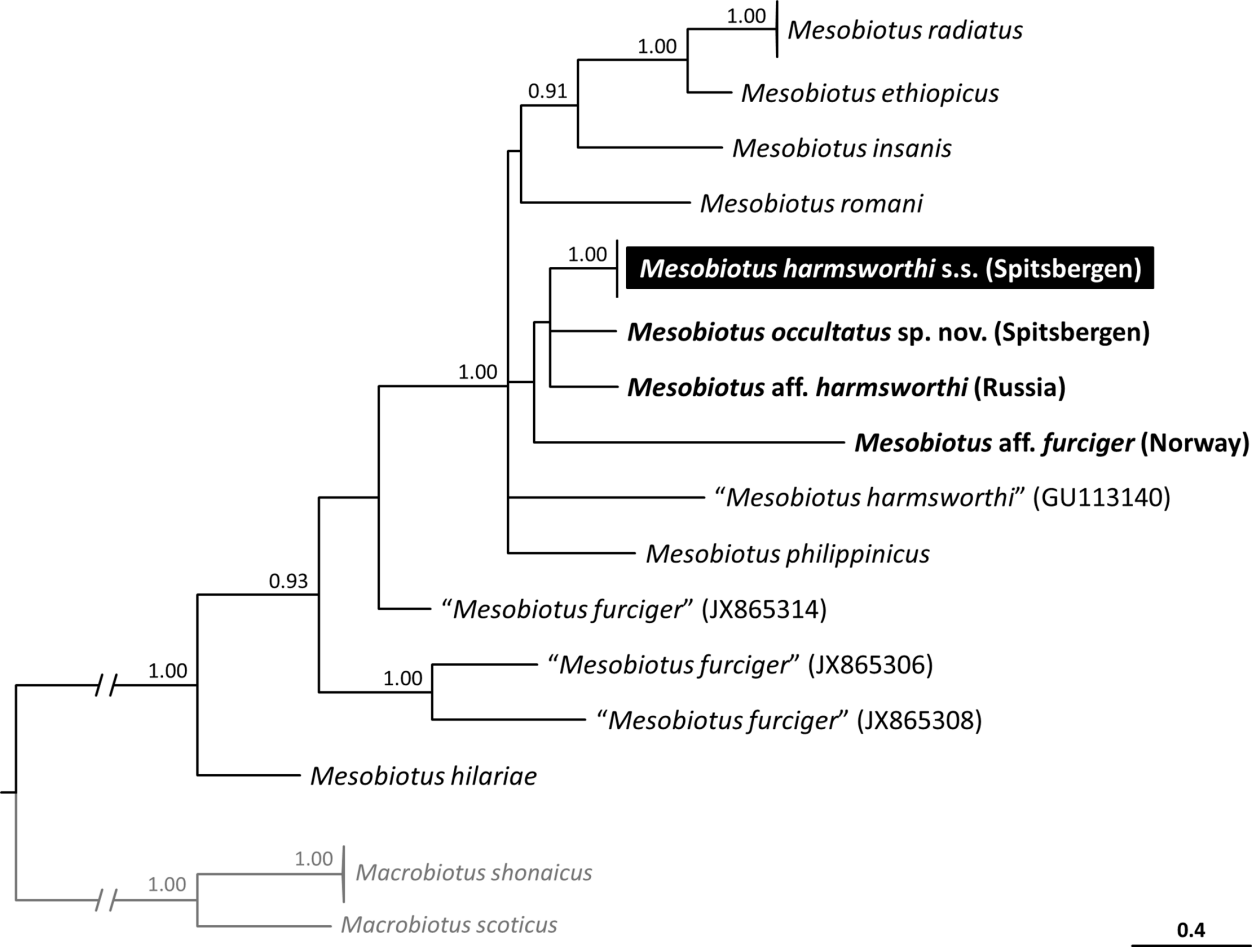
The Bayesian Inference (BI) phylogeny constructed from concatenated sequences (18S rRNA + 28S rRNA + ITS-2 + COI) of the genus *Mesobiotus*. Numbers at nodes indicate Bayesian posterior probability, values below 0.90 are not shown. Please see Table 3 and the “*Phylogenetic analysis*” subsection in Material and Methods for details on species and sequences used in the analysis. Scale bar represents substitutions per position.

The PTP analysis of the COI phylogenetic tree revealed the presence of 14 highly supported species, represented as terminal nodes of ingroup taxa in Fig 18. Although the presented phylogeny was based on four molecular markers, the branch support was generally weak which resulted in some polytomies. Thus, currently, only some preliminary conclusions on the relationships within the genus *Mesobiotus* can be made. The taxa of both species groups recognised within this genus, the *harmsworthi* and the *furciger* group, do not cluster in two evolutionary linages but they are intermixed. *Mesobiotus harmsworthi* redescribed herein is a distinct taxon and, together *M*. *occultatus* **sp. nov.** and an undetermined *M*. *harmsworthi* group species from Russia, the three species form a polytomous clade. The phylogenetic and species delimitation analysis also revealed that an unpublished COI sequence (GU113140) of a specimen from China designated in GenBank as “*M*. *harmsworthi*”, clearly represents a distinct unknown species. Finally, the analysis also showed that three COI sequences of specimens designated in GenBank as “*M*. *furciger*” from three Antarctic islands (JX865306, JX865308 and JX865314), represent three rather than a single species.

## Discussion

### Type and neotype locality of *M*. *harmsworthi*

**Fig 19:**
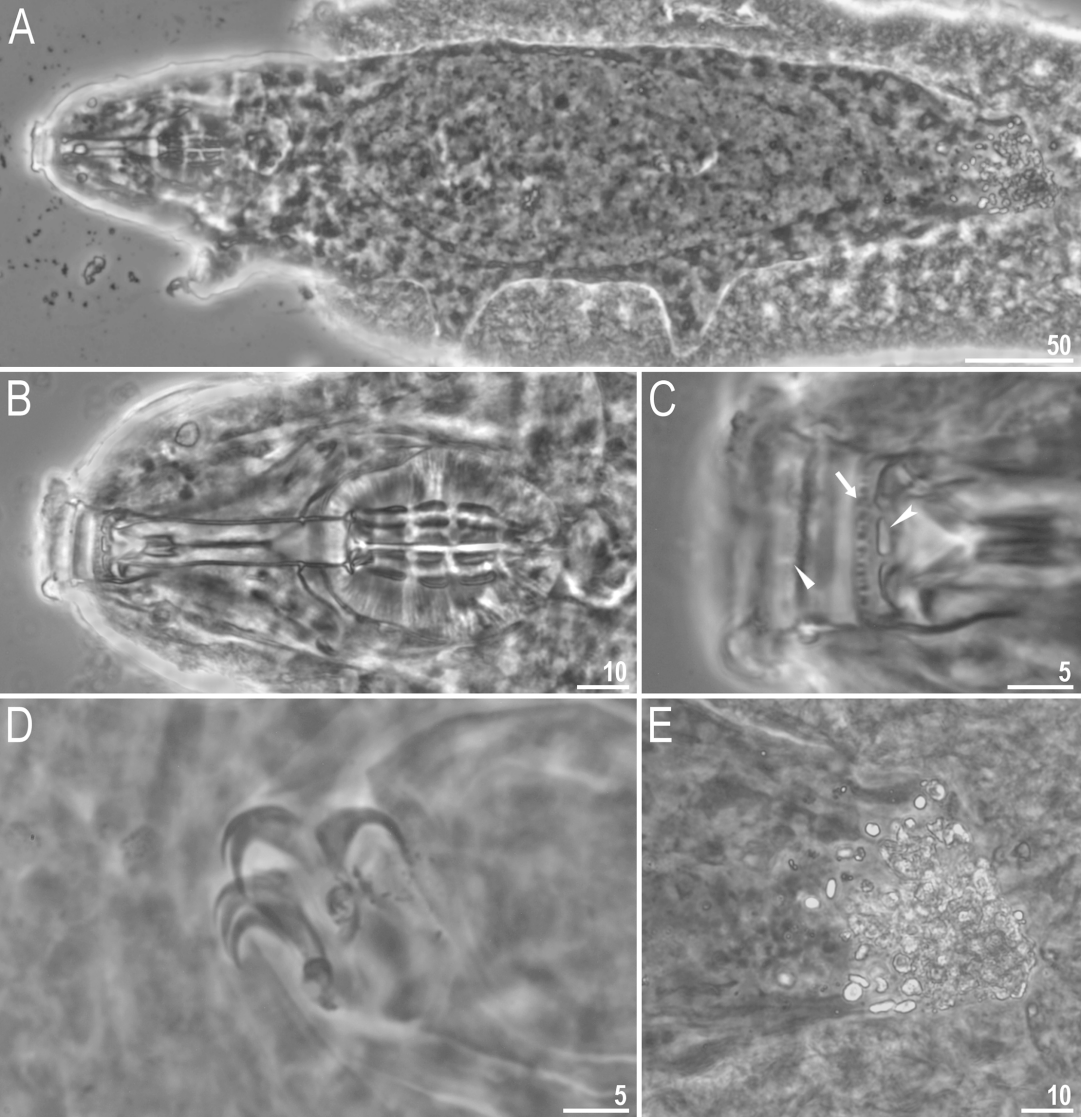
*Mesobiotus* aff. *harmsworthi*—specimen from Shetlands, the Murray collection deposited at the National Museum of Scotland in Edinburgh. A–dorso-ventral projection of the entire animal; B–general view of the bucco-pharyngeal apparatus; C–oral cavity armature; the flat arrowhead indicates teeth of the first band, the arrow indicates teeth of the second band, the indented arrowhead indicates teeth of the third band; D–claws II with smooth lunules; E–caudal part of the body with obscured claws IV (not identifiable). Scale bars in micrometres [μm]. All PCM.

The type locality of *M*. *harmsworthi* is problematic because Murray [11] did not designate a single locale for this species, which was a usual practice in his times, and he stated that he found *M*. *harmsworthi* in Franz Joseph Land, Spitsbergen and Shetland. The first two localities are in the High Arctic and the distance between Cape Mary Harmsworth in Franz Joseph Land and Phippsøya in the Svalbard Archipelago (the most northern locality with a molecularly verified population of *M*. *harmsworthi*) is only *ca*. 435 km. Thus, finding the same species in these two Arctic localities should not be unlikely. The Shetlands, however, are located further south, in a warmer climate zone (Subarctic). Moreover, the distance between Spitsbergen (the most southern locality with a molecularly verified population of *M*. *harmsworthi*) and the Shetland Islands is *ca*. 2000 km. Nevertheless, several tardigrade species were found in localities that were several hundred or a few thousand kilometres apart [74, 75, 76, 77]. However, without the molecular verification, potential cryptic species cannot be ruled out. Moreover, at the time of Murray, the concept of species in tardigrade taxonomy was much different from the current understanding of species. Tardigrade species were thought to exhibit considerable morphological variation and wide (often global) geographic ranges. However, the available integrative data on intra- and interspecific variability, although still scarce, suggest that tardigrade species may exhibit limited morphological variability and geographic distribution, e.g.[76]. Therefore, the presence of *M*. *harmsworthi* in the UK should be treated as a hypothesis rather than as an established fact. This, in turn, implies that Murray’s slides with specimens from the Shetlands, deposited at the National Museum of Scotland in Edinburgh, should not be considered as *M*. *harmsworthi* type material, especially that the name of the species (referring to the Cape Mary Harmsworth) suggests the Arctic to be the primary type locality. Moreover, we examined a specimen deposited at the National Museum of Scotland in Edinburgh, but unfortunately claws IV in that specimen are not visible and it is not possible to verify if the accessory points are in the shape typical for *M*. *harmsworthi* (Fig 19A–19E). Thus, it is not possible to state whether the specimen represents *M*. *harmsworthi* or another species of the *harmsworthi* group. In other words, the specimen should be designated as *M*. aff. *harmsworthi* (see also below).

### Comparison of the neotype with the original description of *M*. *harmsworthi*

The original description of *M*. *harmsworthi* is brief and many taxonomic characters that we currently consider important were described vaguely or were not described at all [11]. Nevertheless, the description states that *M*. *harmsworthi* animals are of a moderate size (up to 500 μm), pale yellow and have very small eyes. The pharynx is oval, with short apophyses, three rod shaped macroplacoids (the third is the longest) and a microplacoid, which is not mentioned in the text, but clearly marked in the drawings. Claws are of the *hufelandi* type, 24 μm long and with large and protruding accessory points on claws IV (also clearly drawn in Fig 7c in [11]; compare with Fig 4B and 4D herein). Lunules under claws I–III are smooth whereas lunulae IV are crenate (dentate). The eggs were not described verbally, but Murray [11] presented a simplistic drawing (Fig 7a in [11]). The number of processes on the circumference of the drawn egg is *ca*. 10–12. Processes are in the shape of sigmoidal cones, with short, narrowed and sharpened apices. The width of process base is similar to process height. Egg processes are sometimes in contact and sometimes they are separated and the morphology of egg surface between the processes is not detailed (Fig 7a in [11]).

The morphology of neoparatypes and additional animals fits the original description well and the trait that distinguishes the species from all other known *harmsworthi* group taxa, the protruding accessory points on hind claws, are clearly marked in the original drawing. The only discrepancy between the description and our redescription is the claw height: whereas the maximal value for the posterior primary branch (including accessory points) in the neotype population was 18.5 μm (with lunules), the original description states 24 μm (in both cases the maximal body length is nearly 500 μm). We suspect, however, that Murray [11] probably measured claws including lunules, which could explain the discrepancy.

In contrast to animals, establishing whether neotype eggs morphology conforms to the type material is more challenging. The original drawing (Fig 7c in [11]) suggests that processes are tightly packed on the egg surface, which does not leave much space for areolation that we observed in the neotype material (Figs 5C and 7A–7D herein). Moreover, the areolation, although often weakly developed, is clearly visible under PCM at least in some *M*. *harmsworthi* eggs. Suspiciously, however, eggs of *M*. *occultatus* **sp. nov.** seem to fit the drawing in [11] much closer than neotype *M*. *harmsworthi* eggs. Processes in *M*. *occultatus* **sp. nov.** are more densely arranged (Fig 11A–11D) and there is no areolation between the processes (Fig 11C and 11D). Moreover, we frequently encountered both *M*. *harmsworthi* and *M*. *occultatus* **sp. nov.** in the same moss samples. Thus, taking all this into consideration, we suspect that Murray [11] most likely linked *M*. *harmsworthi* animals with *M*. *occultatus* **sp. nov.** eggs. If this was indeed the case, we think that animals should be given priority over eggs in establishing which species should be considered *M*. *harmsworthi*. Therefore, individuals exhibiting hind claws with large and protruding accessory points, and with dentate (crenate) lunules should be considered *M*. *harmsworthi s*.*s*. whereas eggs with tightly arranged processes and no areolation should be classified as *M*. *occultatus* **sp. nov.**

### Comparison with other records of *M*. *harmsworthi*

Dastych [15] described *M*. *h*. *obscurus* and differentiated it from *M*. *h*. *harmsworthi* based on the presence of large accessory points, appendices at the bases of egg process and differences in the morphology of the oral cavity armature. Dastych [15] compared his new subspecies with “*M*. *harmsworthi*”, also abundantly represented in his material from Svalbard. He identified individuals and eggs as “*M*. *harmsworthi*” following the characteristics proposed by Argue [78] and the oral cavity armature morphology according to Pilato [79]. However, Argue [78] studied and described specimens from New Brunswick (Canada) and Pilato [79] described the oral cavity of specimens mainly from Italy. Taking into consideration that these specimens were collected far from *loci typici* and from different climate zones, it cannot be ruled out that both Argue [78] and Pilato [79] examined different species of the *harmsworthi* group. In fact, it is not possible to verify their identifications as these authors did not present detailed descriptions of their specimens. Moreover, Argue [78] compared his specimens with the descriptions presented in Marcus [12] and Petersen [80], which are contradictory. Specifically, Marcus [12] described crenate lunules and large accessory points whereas Petersen [80] mentioned that claws were “completely similar to those of *M*. *hufelandi*”, *i*.*e*., with smooth lunules and without protruding accessory points.

The limited original description with a simplistic drawing of the egg allowed to attribute a wide range of animal and egg morphotypes to *M*. *harmsworthi*. In fact, any typical *Mesobiotus* (*Macrobiotus* prior to year 2016) with eggs equipped with conical processes and no areolation on egg surface used to be identified as “*M*. *harmsworthi*” (e.g. see [10]). However, it is clearly visible, from various drawings and/or photos (especially those of eggs) that these probably represent various species of the *harmsworthi* group rather than a single species and most certainly not *M*. *harmsworthi s*.*s*. (e.g. see [4, 12, 78, 81, 82, 83]).

The lack of an accurate diagnosis of *M*. *harmsworthi* resulted in increasing difficulties in the taxonomy of the *harmsworthi* group. Thus, in attempt to clarify the diagnosis of the nominal species for the genus *Mesobiotus*, Pilato et al. [13] analysed animals and eggs from Italy, France and Spitsbergen identified by them “in agreement with tradition” as *M*. *harmsworthi*. Moreover, Pilato et al. [13] examined a slide with an individual identified by Murray as *M*. *harmsworthi*, deposited in the National Museum of Scotland. Importantly, however, it is not clear whether the specimen was designated as a paratype or only a regular specimen that was identified by Murray as *M*. *harmsworthi*. Moreover, it is also not clear if Pilato et al. [13] examined animals or eggs or both from the Shetland Islands. We hypothesise, however, that eggs were not examined because they were not deposited in the National Museum of Scotland. Pilato et al. [13] also presented minimal and maximal values for some morphometric traits of *M*. *harmsworthi* individuals and eggs and provided a drawing of the buccal apparatus, claw and egg, but again, they did not specify which specimens they measured and illustrated. Importantly, hind claws depicted in Fig 3b in Pilato et al. [13] have typical accessory points, which is in disagreement with the original description *M*. *harmsworthi* that clearly states and depicts large accessory points [11]. Moreover, the egg shown in Fig 3c in Pilato et al. [13] has no signs of areolation. This is in contrast to our findings that individuals of *M*. *harmsworthi* exhibit protruding accessory points and lay (at least partially) areolated eggs. Therefore, the drawings in Pilato et al. [13] do not show *M*. *harmsworthi* but a different species of the *harmsworthi* group (i.e. *M*. aff. *harmsworthi*, see below).

The doubts and conflicting characterisations of *M*. *harmsworthi* described above show explicitly that a modern, integrative redescription of the species based on new material from *locus typicus* was very much needed and may allow the discovery of species that otherwise would have been classified as “*M*. *harmsworthi*”.

### Geographic distribution of *M*. *harmsworthi*

Similarly to redescriptions of other nominal taxa with uncertain original diagnoses, e.g. [84, 85], the present redescription of *M*. *harmsworthi* resets the geographic distribution of the redescribed species. Prior to the redescription, “*M*. *harmsworthi*” was reported from all continents [5, 13, 68, 69, 70, 86]. However, comparing neotype material with other records identified as “*M*. *harmsworthi*” (see above for details), we propose that depending on the type of available data, the following identifications may be achieved:

***M*. aff. *harmsworthi***–when individuals and/or eggs fit the general M. harmsworthi morphotype (= an unidentified species of the *M*. *harmsworthi* group).

- ***M*. cf. *harmsworthi***–when qualitative traits fit the redescription but incomplete quantitative data and/or the lack of DNA sequences do not allow a full verification of the record against the neotype series (= a probable record of *M*. *harmsworthi*).
- ***M*. *harmsworthi***–when qualitative and quantitative traits fall within the ranges described in this study and/or DNA sequences show immediate relatedness to the neotype sequences provided here (= a certain record of *M*. *harmsworthi*).

## Conclusions

Based on our analyses, we postulate that all the specimens exhibiting morphology identical with *M*. *h*. *obscurus* should be now considered as the nominal *M*. *harmsworthi s*.*s*., whereas specimens reported from Svalbard by Dastych [15] as *M*. *h*. *harmsworthi* represent *M*. *occultatus* **sp. nov.** The remaining specimens reported as *M*. *harmsworthi* throughout the world should be treated as unidentified species of the *harmsworthi* group (*i*.*e*., as *M*. aff. *harmsworthi*).

## Supporting information

S1 Table Genetic distances. Matrices with calculated uncorrected p-genetic distances.
(XLSX)
